# Multispecies transboundary landscape connectivity in the KAZA TFCA

**DOI:** 10.1038/s41598-026-58440-y

**Published:** 2026-07-06

**Authors:** L. L. Sousa, R. Kotze, L. Fanikiso, K. Young-Overton, J. Overton, X. Stevens, R. Lines, M. Wijers, K. Kesch, D. T. Bauer, J. Seymour-Smith, A. Sibanda, R. Parry, M. Fitt, K. Burger, S. Seyoka, J. W. McNutt, A. B. Stein, V. Pelayo-Malet, L. S. Petracca, P. Funston, P. Henschel, C. W. Winterbach, M. M. Mbizah, S. P. Chatikobo, P. Strampelli, W. A. Nieman, M. J. Claase, D. W. Macdonald, N. Nyambe, A. J. Loveridge

**Affiliations:** 1 Wildlife Conservation Research Unit, Department of Biology, University of Oxford, Oxford, UK; 2 CIBIO, Centro de Investigação em Biodiversidade e Recursos Genéticos, InBIO Laboratório Associado, Campus de Vairão, Universidade do Porto, 4485-661 Vairão, Portugal; 3 BIOPOLIS Program in Genomics, Biodiversity and Land Planning, CIBIO, 4485-661 Vairão, Portugal; 4 Conservation of Natural Ecosystems Trust (CONNECT), Mathiba Road, Maun, Botswana; 5 Panthera, Hook Bridge Camp, Mumbwa GMA, Mumbwa, Zambia; 6 The Nature Conservancy, Plot 377a, House No. 13, Martin Luther King Road, Kabulonga, P.O. Box 51540, Lusaka, Zambia; 7 University of Canterbury at Kent, Kent, CT2 7NR UK; 8 Wildlife Conservation Society, 21, Street 21 Sangkat Tonle Bassac, Phnom Penh, 12000 Cambodia; 9 Victoria Falls Wildlife Trust, Victoria Falls National Park, Victoria Falls, Zimbabwe; 10 Wild Entrust, Botswana Predator Conservation, 520 Mophane Ave., Maun, Botswana; 11 CLAWS Conservancy, Moeti Road, Plot 448, Unit 4, Maun, Botswana; 12 Caesar Kleberg Wildlife Research Institute, Texas A&M University- Kingsville, Howe Agricultural Buildlding #205, Kingsville, TX USA; 13 African Lion Conservation (PTY) LTD, 8 Brabant Close, Marina da Gama, Cape Town, South Africa; 14 Panthera, New York, NY USA; 15 Tau Consultants Pty Ltd, Plot 85, Boro Ward, Maun, Botswana; 16 Wildlife Conservation Action, 1 Alma Close, Strathaven, Harare, Zimbabwe; 17 KAZA Secretariat, President Street, Kasane, Botswana

**Keywords:** Multi-species connectivity, Corridors, Population cores, KAZA, Resistance, Wildlife dispersal areas, Ecology, Ecology, Zoology

## Abstract

This study aimed to delineate multi-species corridors across the KAZA landscape to be included in land use planning and identify which wildlife dispersal areas (WDA) are most critical for seven key mammal species across KAZA. For each species, we modelled habitat suitability across KAZA using ~ 48,000 occurrence points, from both camera trap and spoor surveys. Data from different sources were individually analysed, combined in an ensemble model and final outputs overlayed to form a multi-species layer, with which we assessed connectivity identifying core areas (KDE), corridors (LCP) and functional habitat in KAZA. The central KAZA region, from the Okavango Delta and Chobe National Park to Hwange National Park, supports the most extensive, well-connected core habitat for multiple species. Connectivity weakens toward the Sebungwe region, Angola, Namibia, and Kafue due to major rivers, veterinary fences, and growing human settlement/development. However, substantial highly suitable habitat occurs outside protected areas, with some WDAs playing disproportionately important roles in sustaining species’ connectivity. No single species serves as an umbrella for others when identifying critical corridors, making multi-species analyses essential for comprehensive conservation planning. Protecting corridors and core areas across KAZA depends largely on land uses outside protected areas, underscoring the need to integrate multi-species corridors into land-use planning and to promote coexistence between people and wildlife.

**Supplementary Information:**

The online version contains supplementary material available at 10.1038/s41598-026-58440-y.

## Introduction

Preserving connectivity between protected areas can be critical to conserving large-bodied, migratory and far-ranging mammals^1,2^. Loss of connectivity for these species can have devastating consequences, including inbreeding depression, increased vulnerability to disease, and population collapse^3,4^. For large herbivores and carnivores in Africa, whose space requirements often transcend the borders of formally protected areas, connectivity is vital in sustaining genetically healthy populations^5^. Recognising the long-term conservation importance of facilitating connectivity, which necessitates transboundary agreement^6^, conservation practitioners and governments have developed regional and international treaties to help provide frameworks to promote transboundary collaboration. These include the formal recognition of transfrontier conservation areas (TFCA)^7^ and agreements such as the Convention on Migratory Species^8^. Concurrently, a wide body of work has been developed to identify corridors in these areas that are critical for the maintenance (or re-establishment) of connectivity in the face of human population expansion and associated development^9–11^.

The Kavango–Zambezi Transfrontier Conservation Area (KAZA TFCA) in southern Africa is the world’s largest Transfrontier area, spanning parts of Angola, Botswana, Namibia, Zambia and Zimbabwe, and covering approximately 520,000 km^2^. One of the main objectives driving the establishment of this TFCA was to secure and maintain connectivity for large herbivores and carnivores for ecological benefit, and to provide opportunities for local communities to benefit from tourism and sustainable use^12^. For planning and operational purposes, the TFCA has been strategically divided into wildlife dispersal areas (WDAs) that focus on connecting key protected areas across the landscape^12^. The identification and protection of corridors are central to the WDA strategies, but expansion of human activities and associated infrastructure represents a significant threat to long-term connectivity, particularly because these expansions lack formal spatial planning^12,13^. Where land use plans do exist, many do not formally recognise corridors, and without explicit evidence as to which areas can support the dispersal and migration of large mammals, delineating corridors can be difficult to justify when competing with other land use types^13,14^. To remedy this, the KAZA Secretariat and KAZA Elephant Sub-Working Group developed a Policy Brief for the conservation of elephants (*Loxodonta africana*) across KAZA, a large focus of which was to identify critical pathways to maintain connectivity between KAZA’s National Parks^15^. As elephants are important ecosystem engineers and create distinct pathways across landscapes through repeated use, it was suggested that they could act as a potential umbrella species for connectivity. However, previous multi-species work within KAZA has shown that corridors derived from female elephant movements are not representative of other species^16^. Other multi-species studies have also shown that differences in ecological needs, ranging behaviour, life history strategies and/or trophic guild may limit the degree to which a single species can act as a surrogate for others for corridor allocation^17,18^. There is therefore a need to incorporate data from multiple species into connectivity planning for this TFCA.

Ideally, the identification of corridors would be derived from observation, or telemetry data on species movement across the corridors of interest^9,10,19^. However, the practicality of collecting movement data from individuals making long-distance dispersals (for example through collaring) are low, and building databases that capture these dispersal events is costly and time consuming^19,20^. Given the paucity of sufficient datasets for many species in areas that require connectivity, and the urgency with which conservation decisions need to be made in the face of development^11^, recent advances in modelling have been used to make inferences on how species move through a landscape. Using species presence data, movement data, genetic data or expert opinion, conservation practitioners can determine which landscape features are selected or avoided by the species of interest, and create a continuous surface based on these landscape features^9,21^. Movement data derived from GPS collars have been the gold standard for developing species habitat suitability models (SDMs) and resulting resistance surfaces^9^. However, few long-term datasets contain enough data per species, and less so for multiple species, to reliably inform connectivity modelling across large landscapes and cover context-dependent variation in resource selection that varies with sex, life history stage and seasonality^20,21^. Thus, in the absence of movement data, point data sets are the next most reliable data source^10^ and have been used in both single and multi-species analyses of connectivity^22,23^. Furthermore, point data are generally more widely available geographically, which limits erroneous conclusions drawn across larger landscapes from localised data^24^. The availability of presence-absence data also enables the modelling for multiple species with similar sample sizes.

**Fig. 1:**
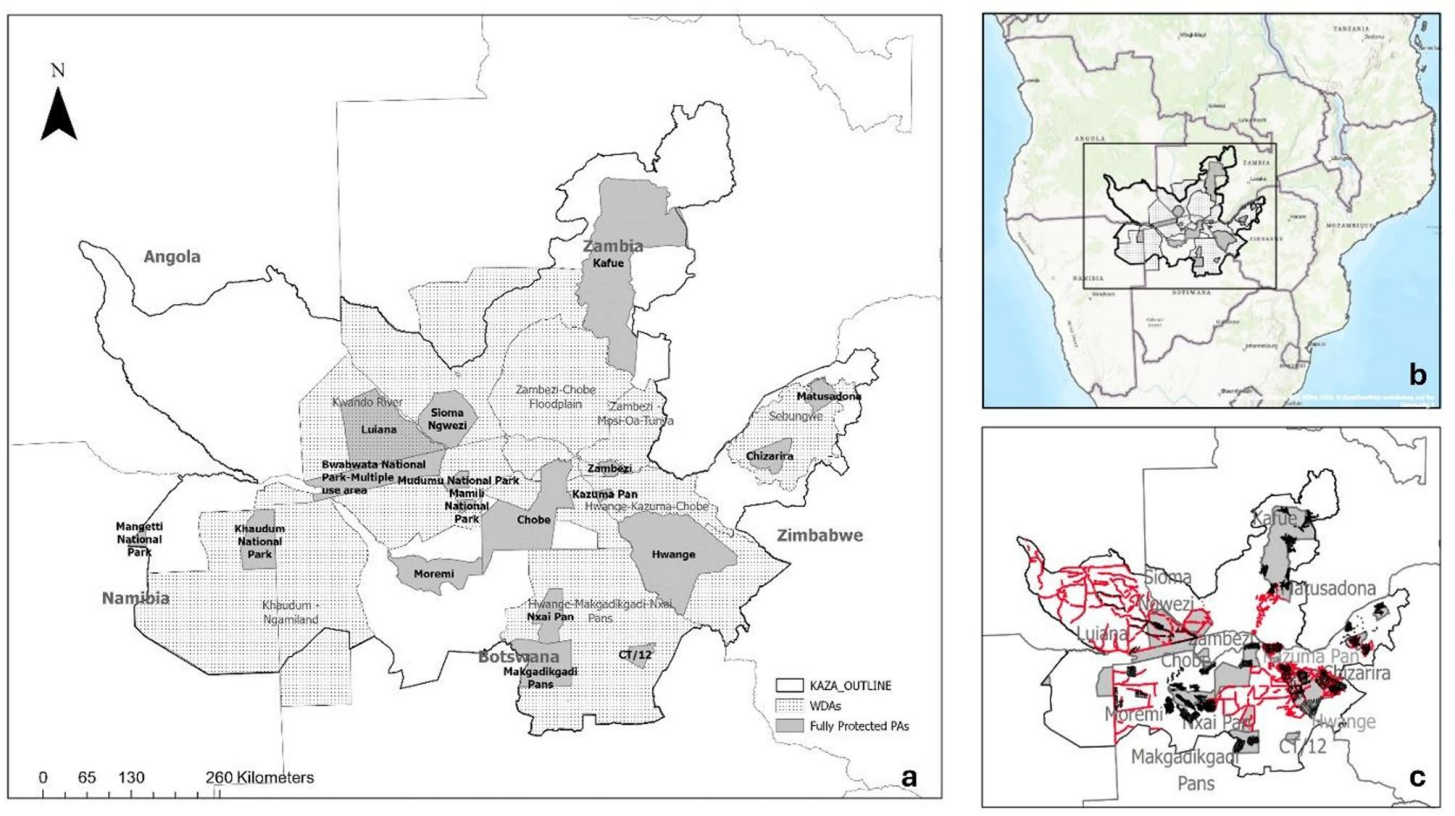
This study focuses on the Kavango–Zambezi Transfrontier conservation area (KAZA) in southern Africa and data was collected from all KAZA partners. Areas in grey represent protected areas (NP = National Park, GR = Game Reserve), dashed areas denote WDAs. The top right panel indicates the positioning of KAZA within southern Africa, and the bottom right panel represents the collected spoor data points (in red) and camera trap data collected (in black).

This study presents a multi-species connectivity analysis across the KAZA landscape (Fig. 1a, b), using a broad detection/non-detection dataset derived from camera trap (CT) and spoor survey efforts in multiple countries and across different land use types (Fig. 1c). The study focuses on four large carnivore and three large herbivore species that have long-distance dispersal or migratory capabilities, namely lion (*Panthera leo*), leopard (*Panthera pardus*), African wild dog (*Lycaon pictus*), spotted hyaena (*Crocuta crocuta*), elephant (*Loxodonta africana*), buffalo (*Syncerus caffer*) and zebra (*Equus burchelli*). Other key species, such as cheetah (*Acinonyx jubatus*) and wildebeest (*Connochaetes taurinus*), were not included due to limited data availability. With the goal of identifying key multi-species corridors across the KAZA WDAs that can be incorporated into land use and conservation planning, this study further highlights WDAs critical for the conservation of multiple and/or specific species. Finally, we also sought to further explore whether any one of the focal species in the analysis could be used as a surrogate or umbrella for connectivity in the context of the multi-species outputs. Maps were created by the authors using ArcGIS Pro 3.5 (Esri, Redlands, CA, USA; https://www.esri.com/en-us/arcgis/products/arcgis-pro).

## Results

Both CT and Spoor datasets generated over 48,000 occurrence records for the species of interest (> 2,700 CT stations of 70 surveys; > 45,500 Spoor 250 m transects, Fig. 1c).

**Fig. 2:**
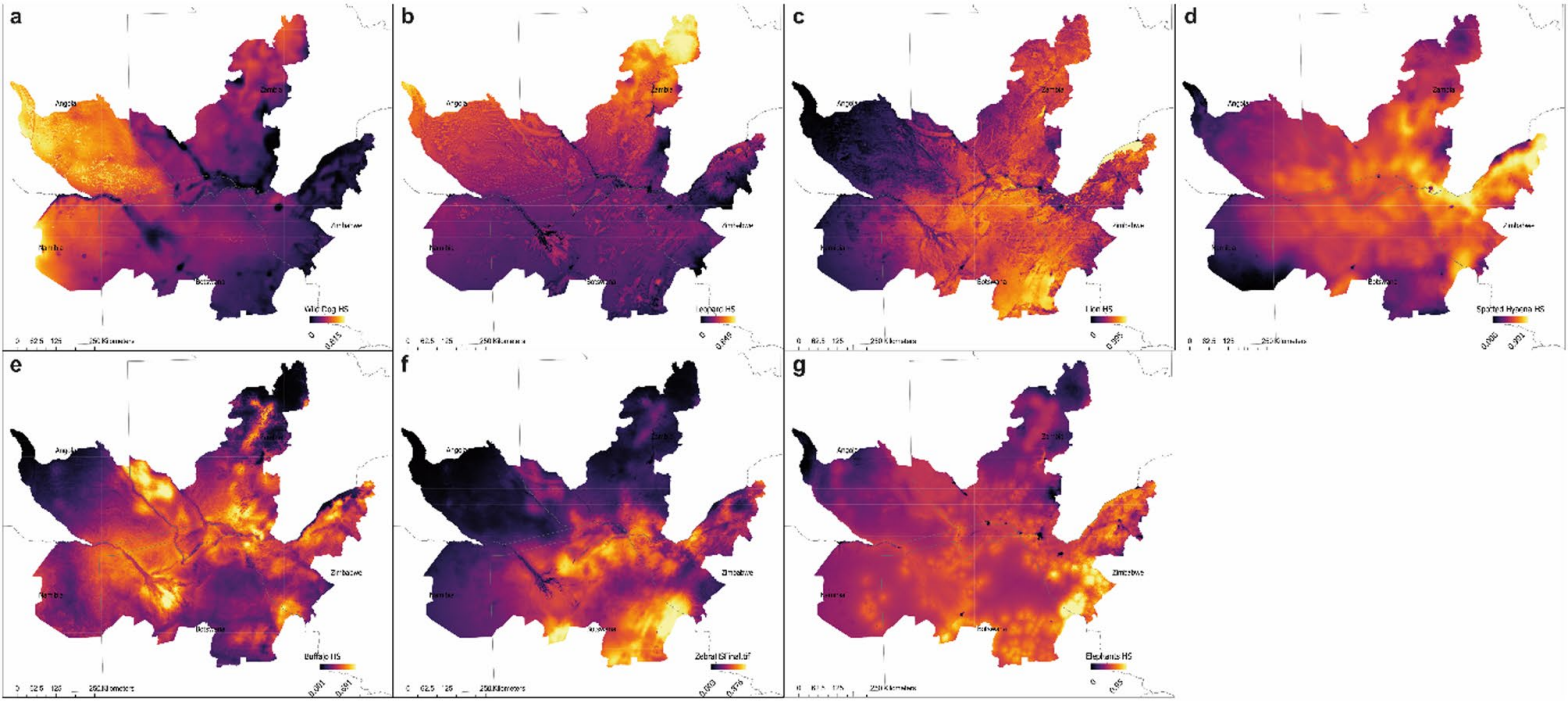
Species-specific habitat suitability ensemble models for (**a**) wild dog (*Lycaon pictus*); (**b**) leopards (*Panthera pardus*); (**c**) lions (*Panthera leo*); (**d**) spotted hyaenas (*Crocuta crocuta*); (**e**) buffalo (*Syncerus caffer*); (**f**) zebras (*Equus quagga*); (**g**) elephants (*Loxodonta africana*). Darker colours identify lower suitability, and warmer colours represent areas of highly suitable habitat respectively.

Having estimated the habitat suitability for each species’ dataset (Fig. 2), both from the camera trap and the spoor surveys, we ensembled the resultant predictive maps using the respective area-under curve AUC (Table 1). The results for each species’ dataset-specific habitat suitability (HS) can be found in the supplementary material (Table S4-S10). In addition, the results from Moran’s I testing and variance partitioning for each model can be found in the supplementary material (Table S11).

**Table 1:** Area-under curve (AUC) values for each species’ habitat suitability model based on the two different datasets. AUC is calculated using the testing dataset per species (30%) and the *performance* function under the ROCR package in R.

Species	Camera traps	Spoor
Lion	0.701	0.622
Leopard	0.698	0.632
Spotted Hyaenas	0.698	0.750
Wild dog	0.642	0.751
Elephants	0.787	0.850
Buffalo	0.749	0.759
Zebra	0.815	0.874

### Multispecies cores and connectivity

We combined the individual cumulative resistance kernel density estimates (KDEs) for all seven species and the least-cost paths (LCP; see Supplementary Figures S2 and S3 for individual species maps) to elucidate multispecies connectivity across KAZA. To highlight these species’ corridors for dispersal, we removed the least likely used corridors for each species defined here as the first quintile of LCP values, and to define the multispecies’ core areas, we selected the top 20th percentile of the averaged KDE (Figs. S2 and S3).

**Fig. 3:**
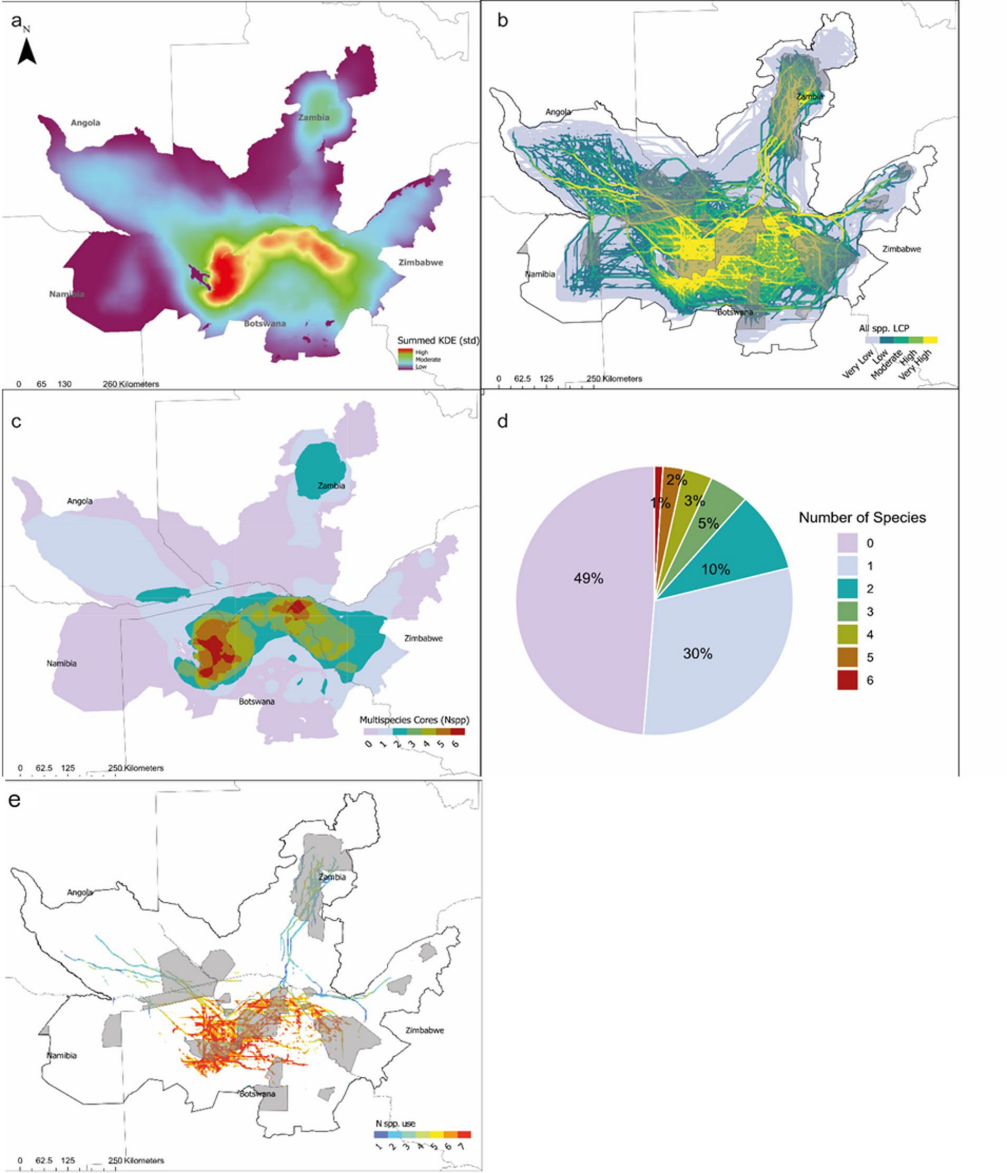
Multispecies connectivity in KAZA. (**a**) Summed multispecies standardised resistant kernel density estimates (KDE) to highlight high intensity used areas; (**b**) summed least cost paths (LCPs), also coloured by intensity of use; (**c**) final core areas as the sum of each species-specific 80th percentile KDE; (**d**) percentage of total KAZA landscape that was identified as core area per number of species, and (**e**) corridors, representing least-cost paths (LCPs) with a higher likelihood of use (> 80% percentile intensity), colour coded by the number of species predicted to use the corridors across the. Species represented Multispecies least cost paths (LCP) with varying probability of use across the Kavango–Zambezi Transfrontier Conservation Area (KAZA TFCA). Multi-species results include wild dog (*Lycaon pictus*), leopards (*Panthera pardus*), lions (*Panthera leo*), spotted hyaenas (*Crocuta crocuta*), buffalo (*Syncerus caffer*), zebras (*Equus quagga*) and elephants (*Loxodonta africana*). Note that all seven species are predicted to mainly overlap at the Hwange-Chobe-Okavango *core* area in central KAZA, with remaining potential high-use corridors being informed by only a few species.

The ensemble model highlighted the connections between Hwange-Chobe-Okavango region stretching from north-western Zimbabwe to northern Botswana, with some linkages to Bwabwata in the Zambezi region of Namibia, to be highly used by most species (Fig. 3a–c). Moreover, this system also has high connectivity potential to the Kafue populations in Zambia. The links to Kafue in Zambia are mainly driven by the connections of lions, spotted hyaenas, buffalo, and elephants (Supplementary Fig. S3–S5). These species also connect to the Sebungwe region of Chizarira and Matusadona National Parks in the far eastern part of KAZA in Zimbabwe. Finally, the strong links to Angola for wild dogs, and to a lesser extent, spotted hyaenas’, are also depicted in the averaged LCP (Supplementary Fig. S3–S5).

Overall, ~ 21% of the KAZA total area is estimated to be a core (high movement density) hotspot for two or more species, whereas only 1% is a core for six species (Fig. 3d). Besides the larger core in central KAZA from the Okavango Delta in Botswana to Hwange protected area system in Zimbabwe, Kafue in Zambia is highlighted as a core area for both lions and leopards, and the north of the Zambezi region in Namibia is mainly defined by the core areas of wild dogs and buffalos (Supplementary Figure S4). Angola and northern Zimbabwe regions are core areas for single species and thus are not represented in our multispecies connectivity map (Fig. 3 and Supplementary Fig. S4).

**Fig. 4:**
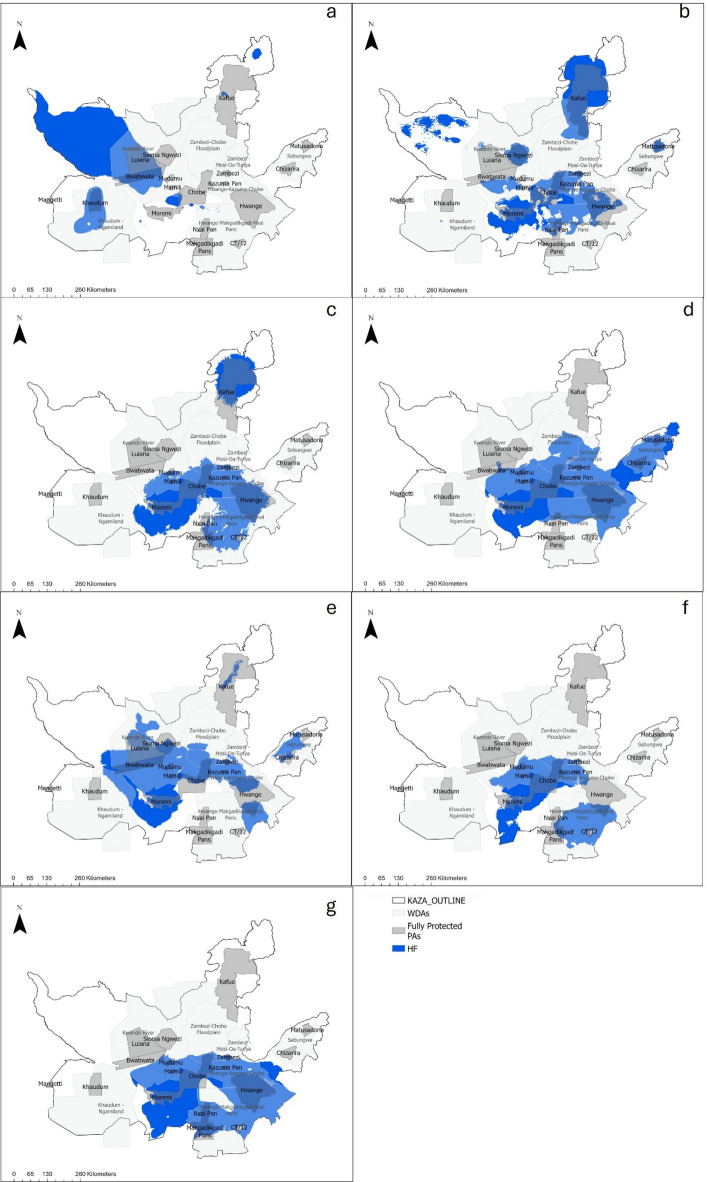
Species specific habitat functionality (HF), wild dog (*Lycaon pictus*), leopards (*Panthera pardus*), lions (*Panthera leo*), spotted hyaenas (*Crocuta crocuta*), buffalo (*Syncerus caffer*), zebras (*Equus quagga*) and elephants (*Loxodonta africana*). Blue denotes HF, grey polygons are Protected Areas (PAs) and shaded areas are Wildlife Dispersal Areas (WDAs).

Our results showed that corridors linking Hwange and Chizarira to the east of KAZA in Zimbabwe; Hwange-Chobe-Okavango core in Zimbabwe and Botswana and Kafue in Zambia; the Hwange-Chobe-Okavango core north-west through Bwabwata (Namibia) into the rest of Angola and between the Chobe-Okavango system south-east to Makgadikgadi Pans NP are all highly used (red links in Fig. 4). Our model predicts several possible, but lower probability of use links between Kafue and the southern system of KAZA, and, additionally, a few lower probability of use corridors link the Sebungwe region and the Hwange system in Zimbabwe. The weakest links appear to be between Makgadigkadi Pans NP and southeast Hwange and the western Okavango Delta in Botswana to Khaudum in eastern Namibia (grey and blue links, Fig. 3e).

Importantly, focusing on the highly used corridors (yellow in Fig. 3b, with an intensity above the 80th percentile), our models reveal the core Hwange-Chobe-Okavango with links to Bwabwata and Makgadikgadi Pans to be intensely used by six and even all seven species (red in Fig. 3e). However, the important links connecting the Sebungwe region, Angola systems and Kafue to the core areas of Botswana and Zimbabwe are only used by up to four species in the analysis (Fig. 3e). Hence, these are likely more fragile corridors that should be granted further protection.

### Surrogacy

Our results show that, for wildlife core areas (KDE) falling outside the Protected Areas (PAs), only leopards and wild dogs do not have the potential to act as an umbrella species (vs. Multispecies) for the other focal species in the analysis (Supplementary Table S13). All other species were found to use the same core areas.

This is not the case when we focus on the corridor level (LCP). In fact, we found that no species has the potential to be classified as a good surrogate for the landscape connectivity of the other six species in analysis, as no species had a higher than 0.65 coefficient between themselves and the multispecies LCP (Supplementary Table S14). Furthermore, we also failed to find a correlation between any pair of single species’ LCP, contrary to what was found for species’ KDEs.

### Conservation value outside protected areas

At the landscape scale, the KAZA protected area (PA) network captured a relatively limited proportion of the most important areas when habitat suitability and connectivity were considered jointly (Fig. 4). Specifically, only ~ 24% of the top 40% habitat functionality (HF) was contained within PAs, while an additional 43% occurred within Wildlife Dispersal Areas (WDAs), highlighting the substantial contribution of multi-use landscapes to maintaining functionally important habitat. Spatial overlap among species within the PA network was limited. Although some areas supported high-value habitat for multiple species, no areas within the PA network simultaneously captured core functional habitat for more than five of the seven species considered. Moreover, approximately 11% of the PA network did not include high-value habitat for any of the focal species. At the species level, the proportion of core functional habitat (top 40% HF) protected within PAs varied markedly. Lions and leopards exhibited the highest representation, with 61% and 64% of their core functional habitat falling within PAs, respectively. In contrast, African wild dogs were poorly represented, with only 19% of their core functional habitat protected. Intermediate values were observed for other species, including spotted hyena (39%), zebra (36%), elephant (41%), and African buffalo (46%), but see Table 2 for detailed results. Overall, these results demonstrate that incorporating habitat functionality reveals important conservation gaps in the current PA network. Large proportions of functionally important habitat occur outside formally protected areas, emphasizing the critical role of WDAs and other unprotected or partially protected landscapes in supporting species persistence and movement across the KAZA region.

**Table 2:** Area and respective percentage of the top 40th percentile of functional habitat for all species combined and then for each of seven species analysed across the Kavango-Zambezi Transfrontier Conservation Area (KAZA TFCA) including wild dog (*Lycaon pictus*), leopards (*Panthera pardus*), lions (*Panthera leo*), spotted hyaenas (*Crocuta crocuta*), buffalo (*Syncerus caffer*), zebras (*Equus quagga*) and elephants (*Loxodonta africana*). The combination of species compares the total area in hectares of the top 40th percentile of all functional habitat for all species inside the protected areas (PA) versus in the wildlife dispersal areas (WDA). Thereafter we compare for each species the percentage of top 40th percentile functional habitat that falls within the protected area (PA) network in KAZA.

Species	Functional habitat—top 40th percentile		
	Combined area in KAZA (km2)	Total area in each category (km2)	Percentage area in each category (%)
All species PA	90,624	21,976	24
All species WDA		39,102	43

Species	Total area in KAZA	Total area inside PA	Percentage inside PA (%)
Wild dog	18,761	3575	19
Lion	20,725	12,660	61
Leopard	20,937	13,426	64
Spotted Hyaena	26,763	10,332	39
Zebra	13,546	4907	36
Elephant	22,291	9121	41
Buffalo	20,219	9318	46

### Wildlife dispersal area (WDA) importance

**Fig. 5:**
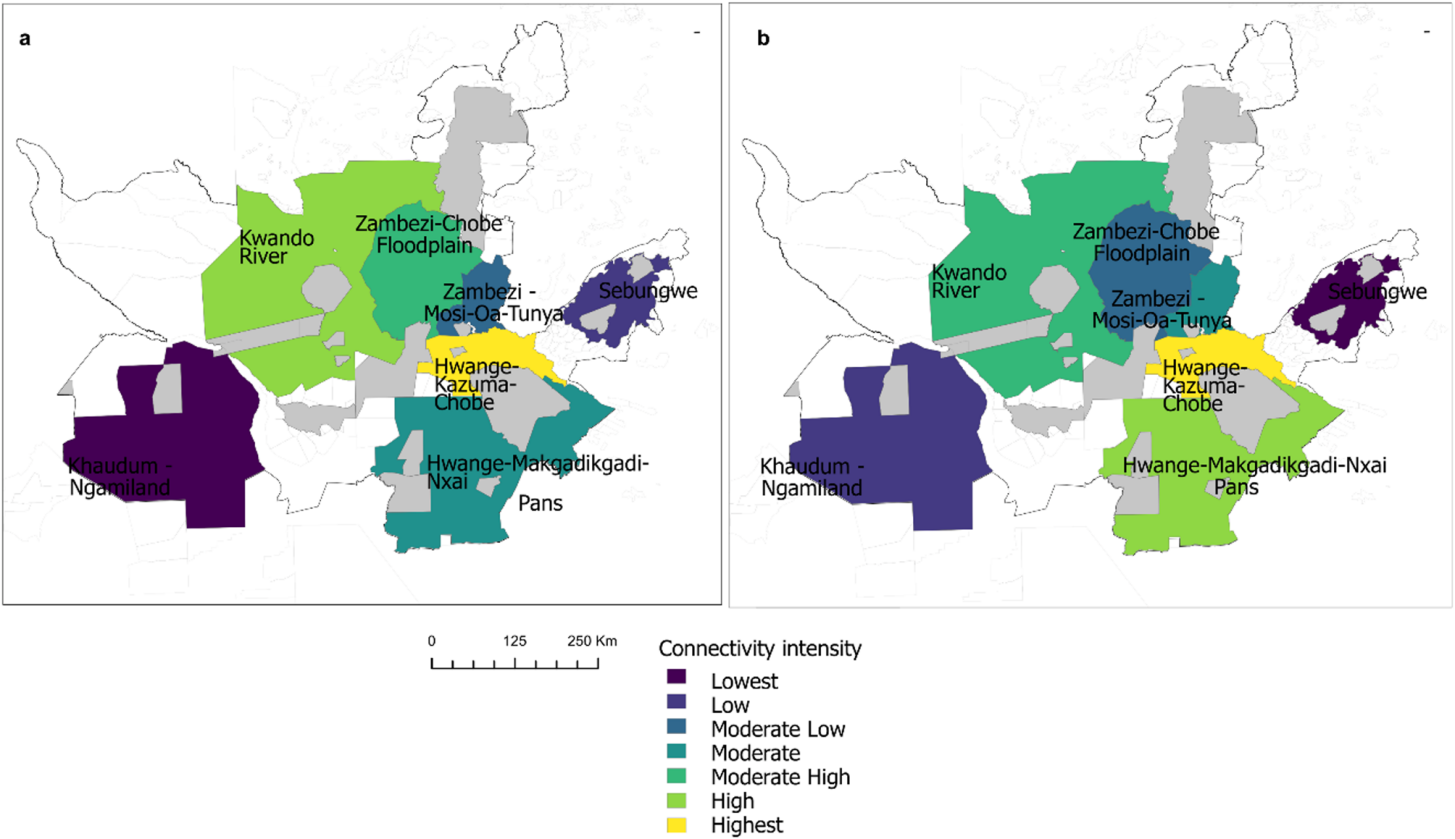
Ranking of Kavango Zambezi Transfrontier Conservation Area (KAZA TFCA) wildlife dispersal areas (WDAs) according to the average intensity of a) potential multispecies dispersal corridors (least-cost paths or LCPs); and b) standardised averaged cumulative resistant kernel density estimates representing core wildlife habitat (KDEs).

When focusing on the connectivity models, and the extent to which each KAZA-designated WDA harbours potential dispersal corridors for the seven species considered in this analysis, we found that the Hwange–Kazuma–Chobe WDA was the KAZA WDA with the highest potential for multispecies connectivity, with highest ranking KDE and LCP values within this WDA (Fig. 5). The LCP distribution outside of the PA network further revealed both the Zambezi-Chobe Floodplain and the Kwando River WDAs as important regions for conservation efforts towards maintaining species connectivity for more than one species. On the other hand, Khaudum-Ngamiland appears to be the WDA with the lower connectivity values for both KDE and LCP (Fig. 5).

## Discussion

In this study, we analysed presence–absence data from spoor and camera-trap surveys, widely used by conservation practitioners for species inventories and population estimates, to develop connectivity models for seven species across extensive and heterogeneous areas of the KAZA landscape. The dataset, comprising more than 48,000 records, represents, to our knowledge, the largest compilation of mammal distribution data assembled for this region. Using both survey types improved our understanding of landscape-scale connectivity and highlighted key data gaps. The resulting resistance-to-movement layers revealed regions that could facilitate connectivity across the landscape while also identifying key hotspots or core habitat areas for each focal species, considering the current size and distribution of source populations for these species. These outputs build on previous work on connectivity in the KAZA landscape^16,25,26^ and help to focus limited conservation funding and guide conservation action for protection of corridors for multiple species within the KAZA WDAs.

Our results estimate that approximately 21% of the total KAZA landscape may potentially function as core habitat for two or more species, whereas only 1% serves as a core area for up to six species, underscoring the importance of a multi-species perspective. Within this limited extent of highly shared core habitat, the most important region spans central KAZA, from the Okavango Delta to the Hwange system. The high connectivity observed in this area is likely linked to the predominance of wildlife-centric land uses across central KAZA. The Moremi Game Reserve and adjacent Chobe National Park in Botswana and Hwange National Park and adjacent hunting blocks to the north in north-western Zimbabwe are all important source populations of our focal species^27,28^. The areas connecting these consist of either privately or community-owned wildlife concessions that focus on consumptive use like hunting, non-consumptive photographic tourism or both. Additionally, central KAZA has the lowest density of people in the TFCA^28^. The lower number of predicted corridors away from this central area to the more peripheral parts of KAZA is likely linked to species-specific traits such as sensitivity to particular barriers, for example, veterinary fences^16,29^, larger rivers and high densities of people. But this could also result from lower species-specific dispersal ability combined with lack of source populations in peripheral parts of the connectivity surface. In the Chobe Zambezi Floodplain region in Zambia and Sebungwe region in Zimbabwe for example, higher densities of people and associated expansion of agriculture and infrastructure are key threats to maintaining connectivity across these areas in KAZA^12^. Integrated land use planning that ensures development and infrastructure do not completely cut off connectivity, and that incorporates measures such as human-wildlife conflict mitigation to ensure the identified multi-species corridors remain permeable to wildlife movement, will be key to securing these connections to the more distal parts of KAZA.

Interestingly, the Angola region, despite not featuring as a multispecies core, still shows a high density of predicted connectivity pathways. This is likely because Angola’s wildlife is still recovering following a decades-long civil war^30^, and so the current densities of species relative to other PAS is low, despite predicted higher habitat suitability for these species across the Luengwe–Luiana and Mavinga PAs. These areas therefore have high recolonization potential from surrounding protected areas that corridor protection could help to facilitate. However, there are several barriers to connectivity in this region. For example, despite higher habitat suitability for elephants and buffalo in this area, the veterinary fences along the Namibian and Botswana border may be one of the key barriers to connectivity into Angola that would allow natural recolonization. Previous studies have shown that these fences impede the movement of buffalo. Additionally, elephant breeding herds cannot cross fences with their calves^16,19^. Higher densities of people and agricultural expansion in the Caprivi strip may block off movement between Botswana, Angola and Sioma Ngwezi NP in Zambia, an issue that will also need to be addressed by land use planning and management^31^.

In contrast, with relatively low numbers of people in the Khaudum Ngamiland WDA, linking the Okavango Delta in Botswana to Khaudum NP in north-eastern Namibia, barriers to connectivity are more likely to be linked to climate. This area is arid and lacks surface water, particularly in the dry season^32^. This observation is supported by our results that show low habitat suitability for water-dependent large herbivores in this region. For carnivores, this also translates into naturally low prey availability, particularly of large prey, limiting large carnivore numbers^32^. Additionally, tolerance for large carnivores is low in this region, leading to high mortality rates for these species—particularly lions^33^. Despite these factors, this region remains the only link between the small resident wildlife populations in Khaudum NP and Nyae Nyae Conservancy in Namibia to the more resource-rich Okavango Delta, with evidence of GPS-collared carnivores and elephants moving through this area^19,33^. As such, this WDA should be prioritised for conservation intervention, with a strong focus on human-wildlife coexistence to limit mortalities of animals moving through the region.

By integrating habitat suitability and connectivity into a single habitat functionality (HF) layer^34,35^, our results provide a more ecologically realistic assessment of conservation performance across the KAZA landscape. This approach moves beyond traditional evaluations based solely on habitat suitability, highlighting not only where species can persist, but also whether those areas are accessible within the broader landscape. At the landscape scale, the relatively low proportion of core functional habitat captured within protected areas (~ 24%) indicates that the current PA network alone is insufficient to maintain ecologically functional landscapes for wide-ranging species. However, the substantial contribution of Wildlife Dispersal Areas (WDAs), which encompass an additional 43% of core functional habitat, underscores the importance of multi-use areas sin supporting connectivity and facilitating movement. This reinforces the view that conservation effectiveness in large transboundary systems such as KAZA depends not only on formally protected areas, but also on the permeability and management of the surrounding landscape matrix. Importantly, the limited spatial overlap of core functional habitat among species suggests that no single set of areas can effectively meet the ecological requirements of all focal species. This has direct implications for spatial planning, indicating that prioritization strategies based on single-species or suitability-only approaches may fail to capture areas that are critical for maintaining multi-species functionality across the landscape. Overall, our results demonstrate that incorporating habitat functionality provides a more comprehensive framework for evaluating conservation performance^36^. By identifying areas that are both suitable and connected, this approach offers a stronger basis for guiding conservation investments, particularly in identifying priority areas outside protected boundaries. Future conservation planning in the KAZA region should therefore explicitly integrate connectivity into decision-making processes, ensuring that both protected areas and the surrounding landscape matrix contribute to sustaining viable and functionally connected populations.

The importance of multi-species connectivity planning in KAZA is further highlighted when examining wildlife dispersal areas (WDAs) in isolation. From a multi-species perspective, corridor use declines toward the more distal parts of the landscape, with fewer species utilising links away from the central Hwange-Okavango core. This spatial variation in connectivity underscores the need to account for multi-species dynamics in conservation planning. This conclusion is further supported by the surrogacy analysis, which shows that no single species adequately represents the connectivity requirements of others, indicating that no species in KAZA can serve as an effective umbrella for landscape connectivity. Apart from differences in dispersal ability, it is likely that the differences in corridors used are also ecologically or anthropogenically driven. Firstly, between guilds, large herbivores are more water-dependent than predators^37,38^ which limits their distribution and connectivity potential to river systems and areas of dry season artificial water provision. Predators are limited by seasonal prey availability, persecution in areas of high human density^39,40^ and intraguild competition^41^. Even within guilds, differences in habitat requirements between generalist and specialist species can result in divergent connectivity needs^16,17^, and our results further support the assertion that using a single umbrella species for connectivity conservation planning is not appropriate for systems where corridors need to support dispersal and migration of multiple species^16^. This variation in connectivity across the KAZA landscape for different species further highlights the importance of heterogeneity over large areas in facilitating the persistence of large mammal guilds. The results for species-specific cores and connectivity in KAZA, and the implications of these are discussed at length in the Supplementary Material.

### Study limitations

While this study uses point data to develop resistance surfaces instead of more favoured but less available movement data^9^, this is advantageous in one way given that presence-absence data is more widely available in many landscapes, is non-invasive, more cost-effective than collecting movement data for multiple species and is thus likely to be repeatable. Furthermore, predicted corridor routes closely match outputs from other single species connectivity models based on movement data (lions^20^, wild dog^26^, elephant^15^) or observed migratory routes for species like zebra in Botswana^42,43^ and dispersal movements of large carnivores in the Khaudum-Ngamiland WDA^33^. This demonstrates the robustness of our single and multi-species results. However, there are limitations to using this type of data too related to model performance where caution needs to be exercised in result interpretation. For example, AUC results were moderate for some species, particularly the lion. Moderate AUC values for some species can be explained by several methodological and ecological factors. First, we used observed absence data rather than pseudo-absences. Although sampling effort was accounted for, variation in the number and reliability of absence records may influence model performance. The number of presence and absences also differed among species, which can affect model accuracy and discrimination ability. Second, model evaluation was based on random partitioning of the data, which does not account for spatial autocorrelation and may lead to optimistic estimates of predictive performance when nearby observations are included in both training and testing sets^44^. Given the spatial structure detected in model residuals, our evaluation metrics should therefore be interpreted with caution. Future work could implement spatially structured cross-validation to provide more robust estimates of predictive performance. However, spatial partitioning can considerably reduce the number of observations available for model calibration and testing within individual folds, particularly for rarer or spatially clustered species, potentially leading to unstable model estimates and inconsistent comparisons among species. We therefore retained a conventional random partitioning framework to ensure methodological consistency across species and modelling stages, while explicitly acknowledging that this approach may produce optimistic estimates of predictive performance in spatially structured datasets. Third, while more flexible algorithms, such as generalised additive models, Random Forest, or MaxEnt, may improve predictive accuracy by capturing nonlinear relationships and interactions, we chose generalised linear models to prioritise interpretability and hypothesis-driven ecological inference. The relative performance of different modelling approaches is context-dependent, and no single algorithm consistently outperforms others across datasets and validation strategies^45–47^. In addition, machine-learning approaches may yield overly optimistic performance estimates when validation data are not fully independent, particularly in spatially structured datasets^45^. Lower AUC values may therefore reflect not only model choice but also ecological characteristics of species, such as broader habitat use, higher mobility, and weaker associations with the environmental predictors included. Although integrated SDMs can strengthen inference by jointly modelling multiple observation processes, in this study, datasets differed substantially in their spatial and temporal coverage, and important detection-related metadata were unavailable across data sources. We therefore adopted a more conventional SDM framework, which we considered the most appropriate and robust approach given the structure and limitations of the available data. Future work could explore more flexible or ensemble modelling approaches to improve predictive accuracy, although this may come at the cost of reduced interpretability and increased model complexity^10,25,26,33^. Species-habitat relationships are inherently spatial because environmental gradients and ecological processes operate across multiple scales^48^. In this study we explicitly evaluated the spatial scale of each predictor to match the scales at which the species likely perceive and respond to their environment. Selecting optimal scales using univariate models does not account for interactions or collinearity among predictors, and the scale identified as optimal in isolation may not remain optimal in a multivariate context, potentially affecting model stability and inference. However, this univariate approach provides a practical way to reduce model complexity and has been widely used in ecological studies^19,41^. Future work could explore multiscale optimisation within a multivariate framework or the use of hierarchical or penalised models to jointly estimate scale effects. We also included sampling effort to control for variation in detectability and site coordinates (X, Y) to account for broad-scale residual spatial dependence. This testing addresses both ecological and statistical aspects of spatial structure, ensuring the relationships we report reflect genuine habitat associations rather than artefacts of sampling bias or unmeasured gradients. Most data were collected in protected areas or areas related to wildlife management or use, precisely because of the need to collect such data for wildlife management and monitoring purposes. However, given that the majority of KAZA is made up of such land use types, we contend this represents a suitable geographical spread across the KAZA landscape. Future studies could focus on increasing data collection in more heavily human populated zones. Due to lack of data sharing permissions we could not acquire data from Namibia in time for analysis. While we did include environmental data from the region representing the current distribution of people, this region is under threat from closing corridors, and the pinch points created in dispersal because of this human expansion may crucially limit connectivity between the core in KAZA and Angola and southwest Zambia^15,23,25,26,33,41,42^. Lastly, it is important to note that all data analysed was dry season data, limiting interference for the wet season. Recent studies have indicated that wet season connectivity analyses can differ substantially from dry season^41^ this is particularly relevant for water-dependent species like zebra, buffalo and elephant whose habitat selection may be largely affected by presence of ephemeral pans and rainfall driven changes in forage availability^33,34,39^. These results should therefore be interpreted as conservative estimates of connectivity^49^. Importantly, analysis of the state of corridors and key habitats should be ongoing. Ensuring the provision of scientific research like this to land use planners and community stewards in KAZA’s non-protected areas is a critical step towards protecting connectivity through improved land use planning that can ensure development for KAZA’s communities while limiting conflict with local wildlife.

## Conclusion

Facilitating the connectivity of both herbivores and large carnivores across the KAZA landscape is likely to be multi-faceted. For large carnivores, connectivity can be fostered principally by limiting conflict with local livestock owners that results in retaliatory killing^50^, and by supporting a prey base outside protected areas. Improving livestock management and care through effective herding and kraaling will likely have a large impact on reducing predation^51,52^, which in turn may improve tolerance of resident carnivores and facilitate safe dispersal of larger predators like lions between protected areas. This is especially critical for species with lower dispersal capability, like leopards and hyaenas, which, due to their generalist habitat selection, are likely to be resident in low densities in the WDAs. It is also critical for wild dogs that are heavily reliant on non-protected areas within KAZA for survival. Creating landscapes of tolerance^50^ towards carnivores is critical for connectivity in KAZA. For large herbivores, veterinary fences are one of the main anthropogenic limits to connectivity in north-western KAZA^16,19^. Negotiations to remove parts of the veterinary fence along the Botswana-Namibia border may be instrumental to the long-term recovery and persistence of large herbivores in south-eastern Angola^29^. Opening these corridors may also be critical for elephants, as facilitating movement to suitable habitat in Angola may relieve the high conflict experienced between people and elephants in northern Botswana.

From a multi-species perspective, two kinds of approaches are clear. For WDAs most under threat, such as the Chobe-Zambezi, Sebungwe, southern Kwando and Mosi-oa-Tunya, where high human habitation may obstruct movement routes, corridor planning at the village level and landscape level will be critical to bridge the gap between micro- and macro-corridor protection respectively^19^. Applying tools like the Land Use Conflict Identification System for micro-corridor planning is one way to achieve this, focusing on specific routes from the model that can be verified with existing indigenous knowledge at local level^53^. Overlaying empirical movement data from other KAZA outputs^16,19,33^ can further help immediate spatial prioritisation of specific corridor routes at the micro-scale, particularly where these routes are imminently threatened by land use change or development^54^. Alternatively, ensemble modelling approaches can be used to combine multiple existing connectivity models to achieve consensus on important wildlife cores, the identification of barriers and agreement on corridors that require zonation and protection mechanisms to secure them to ensure robustness of corridor planning^55^.

Through land use planning exercises, these corridors need to be physically delineated and protected^19^. This would result in the formal recognition of corridors in integrated land use plans along with enabling mechanisms such as conservation agreements with local communities that limit development and encourage wildlife-friendly livelihood practices in the corridors to facilitate wildlife movement. Incentivisation through increased support for human-wildlife conflict in these corridors, the development of wildlife-related economies and resolving of political discrepancies in land allocations will be a necessity for securing these areas and ensuring they are not only corridors on paper^19,31,54^. This incentivisation will need to be carried out at larger scales in areas like Khaudum Ngamiland WDA, central KAZA and the north-eastern parts of the Zambezi Chobe Floodplain WDA, which are less threatened by development and should be managed as connectivity landscapes that maintain permeability for all wildlife species. For both core wildlife area and corridor protection, in KAZA, the involvement of communities in land use planning and upliftment of communities through wildlife friendly livelihoods^56^ will be pivotal in ensuring the continued viability of KAZA as a Transfrontier conservation area that supports connectivity for multiple species.

## Methods

### Data collection and processing

The KAZA secretariat facilitated data-sharing agreements amongst governments and conservation practitioners in the landscape to collect as much information on the target species as possible. Point data in the form of spoor survey and camera trap data were widely available across the landscape (Fig. 1), as these methods are commonly employed by government, NGOs, and research institutions as monitoring tools both inside and outside protected areas. Available data spanned 11 years, from 2013 to 2023. This temporal span allowed for the inclusion of a range of ecological conditions experienced across the KAZA landscape over the last decade, which increased the robustness of the dataset, and given the pace of development was still recent enough to remain relevant. All data collected were from the dry season, spanning from April to October each year, due to the difficulties of data collection in the rainy season.

### Environmental data extraction

Covariates used to develop resistance surfaces were selected based on factors known to influence habitat selection for our target species^16,20,29,42,57,58^.The cloud-based computing platform, Google Earth Engine, was used to access open-source Landsat data to create a time series of dry season (May–October) land cover that spanned 2013–2021 through spectral band calculations. The Enhanced Vegetation Index (EVI) used the near-infrared and red bands to quantify vegetation greenness as a proxy of vegetation productivity. Whilst EVI is a more generalised vegetation index, the Global Forest Cover dataset was used to determine forest extent, which only includes vegetation taller than 5 m. To determine the seasonal variation in water level, a yearly flood inundation layer was calculated in Google Earth Engine through a combination of Short-wave Infrared (SWIR) band thresholding for the semi-arid, desert areas of the TFCA^59^ and a tasselled cap wetness index for the more densely forested areas^60^. Habitat selection was inspected in relation to a KAZA-wide habitat dataset^61^. Finally, we calculated distance to village, town or settlement to be used as a proxy for human pressure, and distance to tarred road or railway to account for the impact of transport infrastructure on animal movement. All datasets were resampled to 250 m resolution to match the transect sections´ resolution of our spoor datasets. Environmental layers processed in Google Earth Engine (GEE) were resampled using a nearest-neighbour approach to maintain the original categorical and discrete pixel values during reprojection and spatial harmonisation. Descriptions of data sources can be found in Table S3 in the supplementary material.

### Species-specific habitat suitability model

Aiming to predict species habitat suitability across KAZA, we processed two distinct datasets collected at different times. Each dataset included the presence and absence records for carnivore and herbivore species (specifically, lions, leopards, wild dogs, spotted hyaenas, buffalo, elephants and zebra) at individual camera stations or transect Sects. (250 m intervals) along the surveyed road (refer to Fig. 1A and B). The dataset included between 280 and 6644 presences depending on the species and data type (see Table S1 in the Supplementary Material). We used observed absences rather than pseudo-absences because sampling was systematic and detection effort was accounted for (via effort in camera traps and number of repeated transect portions sampled in spoor), making absence records more reliable. Consequently, for each species, two distinct models were developed: one utilising the camera trap (CT) data and another using the spoor dataset. Employing a binomial generalised linear model (GLM), for each species’ dataset we partitioned 70% of the presence/absence data for model training, reserving the remaining 30% for model testing. Model performance was evaluated using random partitioning, which may inflate predictive accuracy in spatially structured data^44^. This limitation may partially explain differences in AUC values among species. Furthermore, we used GLMs because they provide interpretable parameter estimates, allow direct hypothesis testing, and are widely used in ecological inference. More flexible approaches (e.g., GAMs, Random Forests, MaxEnt) can improve predictive performance but may reduce interpretability and increase the risk of overfitting. An array of environmental and anthropogenic covariates was then extracted to investigate each species’ specific response to the habitat (refer to Supplementary Table S2 for a comprehensive list of the utilised covariates). These covariates were all scaled and centred. Aiming at more reliable inferences, we developed for each species and dataset a scale optimized habitat suitability model where we tested the influence of each covariate across various spatial scales from fine-resolution local conditions to broad landscape context (250 m, 500 m, 1 k, 2.5 k, 5 k, 7.5 k, 10 k, or 20 k), reflecting the hierarchical nature of habitat selection and the spatial ecology of species^9,58,62^. We did this through the implementation of univariate models per predictor, with the best scale selected based on the Akaike Information Criterion (AIC). This approach is commonly used in multiscale habitat studies but does not account for potential interactions or collinearity among predictors when combined in multivariate models^63^. However, a collinearity test followed the choice of covariates at their retained scale, and any competing models selected by AIC. This approach provides a practical way to reduce model complexity. All analyses were conducted in R (R version 4.1.5; R Core Team, 2022), using the packages *mgcv*^64^*, spdep*^65^*, MuMIn*^66^*, and terra*^67^.

This rigorous approach ensured a comprehensive understanding of each species’ habitat response across multiple datasets and spatial scales. Spatial autocorrelation in species detections is expected, since observations reflect the underlying environmental gradients rather than independent pseudo-replication. Our goal was to describe habitat relationships within the sampled region rather than to extrapolate to new areas further afield. We therefore retained the ecological spatial structure but included site coordinates (X, Y) to account for the residual spatial structure without undermining the ecological signal. Furthermore, we assessed residual spatial autocorrelation using Moran’s I for all models and evaluated the relative contribution of environmental predictors and spatial coordinates using deviance partitioning to ensure that spatial autocorrelation was not the main explanatory driver of observed patterns.

Subsequently, a comprehensive global model was developed by incorporating each predictor at its optimal scale and selecting only the non-collinear set based on a Pearson correlation coefficient threshold of 0.65^68^. The process involved systematically assessing the model’s performance by iteratively excluding each correlated pair of variables and employing the Akaike Information Criterion (AIC) in a stepwise fashion for final model selection. To validate the efficacy of the dataset-specific best model, the AUC was calculated using 30% of the data dedicated to testing.

For each species, we combined both dataset habitat suitability (HS) maps and this ensemble HSspp predicted map resulted from the weighted average based on AUC per data type(AUCct—AUC for camera trap; AUCspoor denotes AUC for the spoor datasets), such as:

*weight SPP CT = AUCct / (AUCct+AUCspoor)*

*weight SPP spoor = AUCspoor / (AUCct+AUCspoor)*

ensemble HS = (weight ct *HSct) + (weight spoor * HS spoor)

### Species-specific connectivity (KDE and LCP)

With the goal of estimating each species’ connectivity across the whole of KAZA, we converted each ensemble HS map into resistance to movements following the negative exponential relationship as per^62^:

R = 100-99*((1-EXP(-coef*HS))/(1-EXP(-coef)))

where R is resistance (ranging from 1 (low landscape resistance to movement) to 100 (high resistance to movement), HS is suitability, and the factor *coef* determines the shape of the relationship. Here, we tested coefficients of 0.05, 0.1, 0.25, 0.5, 0.75, 1, 2, 4, 6, 7 and 12 and selected the coefficient whose regression curve best fitted the relationship between HS and R per species^22^, with coef = 3 for lion, leopard, spotted hyaena and zebra; coef = 4 for elephants, coef = 5 for wild dog and coef = 6 for buffalo. Predictions of current landscape connectivity for all seven species were calculated using cumulative resistant kernels (KDE)^69^ and factorial least-cost paths (LCP)^70^. Source points reflect the distribution and density of the underlying population, with the cost of moving between points or points and pixels in the resistance surface based on the value of the resistance surface. Source points were established from the known distribution and population size of all seven species within KAZA. These were informed by the most up-to-date estimates from various aerial survey accounts and predator censuses across the region^28,71–86^, with expert opinion or unpublished survey results from local NGOs filling in the gaps. Source points equivalent to the number of individuals estimated to be in each area were generated randomly within polygons depicting the extent of each area with total numbers shown in Supplementary Material Table S2. Within each subpopulation polygon, for which species-specific densities are known, spatial points (representing source or destination locations for dispersing animals) were randomly distributed at densities proportional to population size.

We used UNICOR^87^ to calculate the KDE and LCP density maps. The KDE model calculates the expected movement of dispersing animals per pixel of the resistance surface, given the dispersal ability of the species (see Supplementary Material Table S12), the density and distribution of the source population and the resistance of the landscape^69^ and can be interpreted as the probability of a dispersing animal traversing each pixel. The LCP analysis calculates the least-cost paths among all combinations of source points and sums them to create a path density map reflecting the relative strength of linkages across the network^88^. The LCP approach complements the KDE by defining corridors between core habitat patches in the landscape and potential constraints to dispersal movement patterns^89^. We calibrated the cost unit thresholds for the LCP against the known dispersal capacity of each species (Supplementary Table S10). Following previous studies, the LCP network is calculated with 4–5 × the cumulative resistance kernels thresholds to simulate the long-range dispersal of individuals^25,90^.

### Multispecies connectivity

In order to identify the landscape areas prone to facilitate the connectivity of the focal group of species, and how the dispersal of these is shaped across KAZA, we then merged the individual maps to design a multispecies cumulative kernel and movement corridors. Firstly, we standardised the resistant KDE and LCP, per species, into 0–1 maps, with 1 denoting the highest connectivity for each species, and then averaged the resultant maps to inform on the landscape connectivity across the seven species. To highlight *core areas* of connectivity for all species in the region, we classified the averaged KDE into percentiles and assigned a *core* to those regions with kernel values above the 80th percentile.

To highlight the areas with a higher likelihood of use by the seven species in analysis, as a proxy for broader corridor planning, we first standardised individual species’ least-cost paths, after removing the weakest, least likely used paths (below the 20th percentile), and reclassified each species’ LCP into ‘1’. We finally summed these binary maps and classified the sum into five categories of use intensity, depicting the corridors with higher predicted usage by our models. To depict those paths with high use both at the intensity and the number of species using them, we clipped the most likely used corridors (above 80% of predicted usage) and summed the binary individual species corridors that composed these.

### Surrogacy

We investigated each species’ connectivity umbrella potential by assessing the correlation between multispecies connectivity maps (based on averaged KDE and LCP outputs) and corresponding single-species maps. Such approaches are commonly used to evaluate surrogate or umbrella species effectiveness in conservation planning^18,91,92^. Pearson correlation coefficients were calculated using the *Band Collection Statistics tool* in ArcGIS to quantify the similarity between spatial predictions. A threshold of r ≥ 0.5 was used to indicate moderate to strong positive correlation, following conventional interpretations of effect size, where values above 0.5 are generally considered to reflect substantial agreement between variables.

### Conservation value outside protected areas

To better capture the spatial interplay between habitat suitability and landscape connectivity, we developed an integrated layer of habitat functionality following recent approaches^34,35^. Habitat functionality was defined as the spatially explicit combination of species-specific habitat suitability (HS) and connectivity (KDE), thereby identifying areas that are both suitable for the species and accessible within the landscape. The two layers were rescaled to comparable ranges, and habitat functionality was then calculated by integrating suitability and connectivity values at each raster cell. Specifically, *HF* = *Suitability* × *Connectivity*, emphasising areas where both suitability and connectivity are high, while down-weighting locations where either component is limiting. The resulting habitat functionality layer was used to (i) identify key areas supporting both species persistence and movement, and (ii) evaluate the performance of protected areas (PAs) in capturing functionally important habitat. For each species, we quantified the proportion of high-functionality areas within current protected area systems in KAZA (defined in this context as gazetted National Parks and Game Reserves within the KAZA landscape whose primary role is wildlife preservation and allows only non-consumptive tourism). We used HF instead of core connectivity, as this could also represent recovery potential for species in areas where habitat is suitable but is currently not at high densities. Hence, we calculated the percentage of HF of each species within the PA network and, highlighting the areas predicted to be important (e.g., habitat functionality) that fall outside the current systems of protected areas, we calculated, per focal species, within each of the designated WDAS in KAZA, a) the percentage of HF. Finally, we also calculated the average LCP intensity using the combined multispecies LCP map to illustrate the importance of each area in maintaining species connectivity that is not under PA protection.

## Supplementary Information

Below is the link to the electronic supplementary material.

Supplementary Material 1.

Supplementary Material 2.

Supplementary Material 3.

Supplementary Material 4.

## Data Availability

The datasets generated during and/or analysed during the current study are available as Supplementary material.
