# Supplementary material for: Multispecies transboundary landscape connectivity in the KAZA TFCA
Source: Sci Rep. 2026 Jul 6;16:24330. doi: 10.1038/s41598-026-58440-y (PMC13447834; doi:10.1038/s41598-026-58440-y)
Supplement: Supplementary file 3 [file 41598_2026_58440_MOESM4_ESM.docx]

**SUPPLEMENTARY MATERIAL**

Table S1. Presence and absence records for seven species, namely wild dog (*Lycaon pictus*, leopard (*Panthera pardus*), lion (*Panthera leo*), spotted hyaena (*Crocuta crocuta*), buffalo (*Syncerus caffer*), zebra (*Equus quagga*) and elephant (*Loxodonta africana*) from camera trap and spoor surveys in the Kavango Zambezi Transfrontier Conservation Area (KAZA TFCA).

| Species | Presence | Absence | Total | % Presence |
| --- | --- | --- | --- | --- |
| Camera Trap Data | | | | |
| Buffalo | 764 | 1947 | 2711 | 28% |
| Elephant | 2189 | 522 | 2711 | 81% |
| Leopard | 1454 | 1257 | 2711 | 54% |
| Lion | 910 | 1801 | 2711 | 34% |
| Spotted hyaena | 1937 | 774 | 2711 | 71% |
| Wild dog | 585 | 2126 | 2711 | 22% |
| Zebra | 919 | 1792 | 2711 | 34% |
| Spoor data | | | | |
| Buffalo | 822 | 44742 | 45564 | 2% |
| Elephant | 6644 | 38920 | 45564 | 15% |
| Leopard | 1477 | 44087 | 45564 | 3% |
| Lion | 721 | 44843 | 45564 | 2% |
| Spotted hyaena | 3584 | 41980 | 45564 | 8% |
| Wild dog | 280 | 45284 | 45564 | 1% |
| Zebra | 1619 | 43945 | 45564 | 4% |

**Table S2 Estimated N per species within KAZA which informed the number of source points to be generated for connectivity modelling.**

|  | Buff | WD | Spott | Lion | Leop | Eles | Zebra |
| --- | --- | --- | --- | --- | --- | --- | --- |
| Est. N | 70500 | 1754 | 14110 | 1999 | 6163 | 227800 | 75700 |

***Species Specific Habitat Suitability Modelling***

Table S3. List of all the covariates used to develop species habitat suitability models for the seven focal species across the Kavango Zambezi Transfrontier Conservation Area, including wild dog (*Lycaon pictus*), leopard (*Panthera pardus*), lion (*Panthera leo*), spotted hyaena (*Croctua crocuta*), buffalo (*Syncerus caffer*), zebra (*Equus burchelli*) and elephant (*Loxodonta africana*).

| **Covariate** | **Description** | **Source** |
| --- | --- | --- |
| Rainfall | Average annual rainfall (unit of measurement) per 1km pixel size | ^1^ |
| Soil carbon | Total organic soil carbon (above ground and below ground) as a proxy for ecosystem and land productivity. | ^2^ |
| Tree cover | Percentage tree cover per pixel, used as a proxy for available cover (1-100%) | ^3^ |
| Permanent water | Proportion of area covered by water (0-1) as well as distance to water (km) | ^4,5^, calculated in Google Earth Engine |
| EVI | Enhanced Vegetation Index, used as a proxy for vegetation productivity (RANGE) | Landsat 8, calculated in Google Earth Engine |
| Habitat type | Proportion of habitat type (Bushland, Cropland, Floodplain, Open Shrubland, Open woodland, Woodland Dense) per pixel | ^6^ |
| Effort | Camera trap datasets: number of active cameras trap nights per stations  Spoor datasets: number of temporal repeats of each 250m segment covered | Generated from each dataset |
| Distance to nearest Village | Distance to nearest town, village or settlement | https://data.humdata.org/ |
| Roads, Railway | Distance to major tarred roads or railways throughout the KAZA landscape | https://data.humdata.org/ |
| Fence | Distance to veterinary cordon fence | KAZA Dataset |

***Results***

***Species specific habitat suitability models***

The results from species-specific habitat suitability models are included below (Table S4-Table S9).

Table S4. Habitat suitability model results showing variable coefficients for buffalo (*Syncerus caffer*) presence data derived from camera trap (CT) and spoor datasets from across the Kavango Zambezi Transfrontier Conservation Area (KAZA TFCA). Details on model fits and combining datasets for ensemble habitat suitability maps are provided in the main text. Table S1 outlines the variables used for modelling. Variables followed by a number indicate the scale at which that particular variable was most influential ranging from 250m, 500m, 1k, 2.5k, 5k, 7.5k, 10k, or 20k.

| CT | | | | |
| --- | --- | --- | --- | --- |
| Coefficients: | |  |  |  |
|  | Estimate | Std. | Error | z |
| (Intercept) | -1.93368 | 0.204978 | -9.434 | < 2E-16 |
| scale(x) | -0.57858 | 0.102598 | -5.639 | 1.71E-08 |
| scale(y) | 0.845691 | 0.161275 | 5.244 | 1.57E-07 |
| scale(DistFence) | -1.2081 | 0.162813 | -7.42 | 1.17E-13 |
| scale(evi_2050) | 0.393378 | 0.073278 | 5.368 | 7.95E-08 |
| scale(FP_20k) | 0.373107 | 0.085529 | 4.362 | 1.29E-05 |
| scale(Crop_500) | -0.15693 | 0.101591 | -1.545 | 0.122 |
| scale(DistWater) | -0.41628 | 0.098928 | -4.208 | 2.58E-05 |
| Effort | 0.014927 | 0.003638 | 4.103 | 4.08E-05 |
|  |  |  |  |  |
| SPOOR | | | | |
| Coefficients: | |  |  |  |
|  | Estimate | Std. | Error | z |
| (Intercept) | -4.78808 | 0.09412 | -50.874 | < 2E-16 |
| scale(x) | 0.37612 | 0.1213 | 3.101 | 0.00193 |
| scale(y) | 0.37603 | 0.09544 | 3.94 | 8.15E-05 |
| scale(WoodlDen_7) | -0.32542 | 0.16254 | -2.002 | 0.045275 |
| scale(TC__10k_) | -0.39241 | 0.08377 | -4.685 | 2.81E-06 |
| scale(Human20) | 0.10063 | 0.02857 | 3.522 | 0.000428 |
| scale(evi20k) | 0.24651 | 0.05838 | 4.222 | 2.42E-05 |
| scale(FP_20k) | -0.76882 | 0.16264 | -4.727 | 2.28E-06 |
| scale(Water_5) | 0.25442 | 0.02651 | 9.598 | < 2E-16 |
| scale(Crop_20) | 0.05605 | 0.03698 | 1.516 | 0.129596 |
| scale(OWL_10) | 0.26487 | 0.05578 | 4.749 | 2.05E-06 |
| scale(RoadDens500m) | -0.18492 | 0.05768 | -3.206 | 0.001345 |
| scale(DistWater) | -0.74691 | 0.16075 | -4.646 | 3.38E-06 |

Table S5. Habitat suitability model results showing variable coefficients for elephant (*Loxodonta africana*) presence data derived from camera trap (CT) and spoor datasets from across the Kavango Zambezi Transfrontier Conservation Area (KAZA TFCA). Details on model fits and combining datasets for ensemble habitat suitability maps are provided in the main text. Table S1 in the outlines the variables used for modelling. Variables followed by a number indicate the scale at which that particular variable was most influential ranging from 250m, 500m, 1k, 2.5k, 5k, 7.5k, 10k, or 20k.

| CT | | | | |
| --- | --- | --- | --- | --- |
| Coefficients: | |  |  |  |
|  | Estimate | Std. | Error | z |
| (Intercept) | 0.76354 | 0.2205 | 3.463 | 0.000535 |
| scale(x) | -0.37434 | 0.12545 | -2.984 | 0.002844 |
| scale(y) | -0.81756 | 0.09661 | -8.463 | < 2E-16 |
| scale(Rain500) | -0.19204 | 0.09257 | -2.074 | 0.038037 |
| scale(Human2_5) | -0.664 | 0.08504 | -7.808 | 5.79E-15 |
| scale(evi_2050) | -0.26354 | 0.07754 | -3.399 | 0.000676 |
| scale(FP_20k) | 0.69648 | 0.15457 | 4.506 | 6.61E-06 |
| scale(DistWater) | -0.84735 | 0.11165 | -7.59 | 3.21E-14 |
| Effort | 0.02401 | 0.00402 | 5.972 | 2.34E-09 |
|  |  |  |  |  |
| SPOOR | | | | |
|  | Estimate | Std. | Error | z |
| (Intercept) | -3.22822 | 0.06098 | -52.939 | < 2E-16 |
| scale(WoodlDe_10) | -0.77826 | 0.07101 | -10.96 | < 2E-16 |
| scale(Bush_500) | 0.09317 | 0.02016 | 4.622 | 3.80E-06 |
| scale(OWL_20) | 0.4647 | 0.01985 | 23.41 | < 2E-16 |
| scale(TC__500k) | -0.21939 | 0.03005 | -7.301 | 2.86E-13 |
| scale(Water_20) | 0.04761 | 0.01286 | 3.702 | 0.000214 |
| scale(FP_20k) | -0.39316 | 0.0497 | -7.911 | 2.56E-15 |
| Effort | 0.0784 | 0.02096 | 3.739 | 0.000184 |
| scale(evi20k) | 0.35012 | 0.02401 | 14.583 | < 2E-16 |
| scale(Carbon20_1) | 0.065 | 0.0326 | 1.994 | 0.046191 |
| scale(Human500) | -0.30478 | 0.02729 | -11.169 | < 2E-16 |
| scale(DistVill) | 0.11102 | 0.02072 | 5.359 | 8.39E-08 |
| scale(DistWater) | -1.60651 | 0.06789 | -23.663 | < 2E-16 |
| scale(DistFence) | -0.47875 | 0.06522 | -7.341 | 2.13E-13 |

Table S6. Habitat suitability model results showing variable coefficients for zebra (*Equus burchelli*) presence data derived from camera trap (CT) and spoor datasets from across the Kavango Zambezi Transfrontier Conservation Area (KAZA TFCA). Details on model fits and combining datasets for ensemble habitat suitability maps are provided in the main text. Table S1 outlines the variables used for modelling. Variables followed by a number indicate the scale at which that particular variable was most influential ranging from 250m, 500m, 1k, 2.5k, 5k, 7.5k, 10k, or 20k.

| CT | | | | |
| --- | --- | --- | --- | --- |
| Coefficients: | |  |  |  |
|  | Estimate | Std. | Error | z |
| (Intercept) | -2.119 | 0.207 | -10.257 | < 2E-16 |
| scale(x) | 0.050 | 0.112 | 0.443 | 0.657942 |
| scale(y) | -0.506 | 0.132 | -3.824 | 0.000131 |
| scale(Rain20k) | -0.195 | 0.096 | -2.036 | 0.04177 |
| scale(TC500k) | -0.402 | 0.141 | -2.852 | 0.004341 |
| scale(Carbon7_5k) | -0.156 | 0.0909 | -1.748 | 0.080499 |
| scale(FP_20k) | 0.166 | 0.101 | 1.642 | 0.100688 |
| scale(Crop_10) | -0.184 | 0.080 | -2.298 | 0.021581 |
| scale(OWL_500) | -0.202 | 0.083 | -2.443 | 0.014546 |
| scale(OpenSh_3) | -0.145 | 0.110 | -1.323 | 0.185991 |
| scale(Bush_20k) | -0.215 | 0.096 | -2.244 | 0.024855 |
| scale(Water_1) | -0.672 | 0.156 | -4.317 | 1.58E-05 |
| scale(DistWater) | -0.806 | 0.116 | -6.948 | 3.70E-12 |
| Effort | 0.013 | 0.004 | 5.193 | 2.07E-07 |
|  |  |  |  |  |
| SPOOR | | | | |
| Coefficients: | |  |  |  |
|  | Estimate | Std. | Error | z |
| (Intercept) | -7.612 | 0.227 | -33.487 | < 2E-16 |
| scale(x) | 1.038 | 0.113 | 9.196 | < 2E-16 |
| scale(y) | 0.047 | 0.101 | 0.467 | 0.64069 |
| scale(WoodlDe_12) | -0.265 | 0.162 | -1.636 | 0.10193 |
| scale(rain20k) | -1.159 | 0.086 | -13.446 | < 2E-16 |
| scale(TC__500k) | -0.258 | 0.051 | -5.047 | 4.49E-07 |
| scale(Carbon7__1) | -0.267 | 0.057 | -4.646 | 3.38E-06 |
| scale(evi20k) | 0.903 | 0.0628 | 14.505 | < 2E-16 |
| scale(FP_20k) | -1.339 | 0.148 | -9.078 | < 2E-16 |
| scale(OWL_20) | 0.633 | 0.055 | 11.528 | < 2E-16 |
| scale(Bush_500) | -0.130 | 0.047 | -2.742 | 0.00611 |
| scale(DistWater) | -4.249 | 0.218 | -19.507 | < 2E-16 |
| scale(DistFence) | -0.688 | 0.166 | -4.145 | 3.40E-05 |

Table S7. Habitat suitability model results showing variable coefficients for spotted hyaena (*Crocuta crocuta*) presence data derived from camera trap (CT) and spoor datasets from across the Kavango Zambezi Transfrontier Conservation Area (KAZA TFCA). Details on model fits and combining datasets for ensemble habitat suitability maps are provided in the main text. Table S1outlines the variables used for modelling. Variables followed by a number indicate the scale at which that particular variable was most influential ranging from 250m, 500m, 1k, 2.5k, 5k, 7.5k, 10k, or 20k.

| CT | | | | |
| --- | --- | --- | --- | --- |
| Coefficients: | |  |  |  |
|  | Estimate | Std. | Error | z |
| (Intercept) | -0.539 | 0.191 | -2.826 | 0.004718 |
| scale(Rain5k) | -0.761 | 0.106 | -7.176 | 7.19E-13 |
| scale(DistFence) | 0.177 | 0.118 | 1.502 | 0.133138 |
| scale(Human2_5) | -0.41 | 0.090 | -5.250 | 1.52E-07 |
| scale(Carbon7_5k) | 0.174 | 0.079 | 2.209 | 0.027157 |
| scale(evi_2050) | -0.259 | 0.071 | -3.667 | 0.000245 |
| scale(FP_20k) | 0.372 | 0.1263 | 2.949 | 0.003186 |
| scale(Water_20) | -0.124 | 0.077 | -1.617 | 0.105898 |
| scale(OWL_20) | 0.578 | 0.146 | 3.963 | 7.41E-05 |
| scale(OpenSh_4) | 0.204 | 0.152 | 1.342 | 0.179699 |
| scale(Bush_7_5) | 0.375 | 0.093 | 4.055 | 5.01E-05 |
| scale(WoodlDen_4) | -0.270 | 0.078 | -3.479 | 0.000503 |
| scale(DistWater) | -0.826 | 0.087 | -9.502 | < 2E-16 |
| scale(DistVil) | 0.149 | 0.077 | 1.933 | 0.053218 |
|  |  |  |  |  |
| SPOOR | | | | |
| Coefficients: | |  |  |  |
|  | Estimate | Std. | Error | z |
| (Intercept) | -3.056 | 0.040 | -75.517 | < 2E-16 |
| scale(x) | 0.465 | 0.055 | 8.438 | < 2E-16 |
| scale(y) | 0.152 | 0.051 | 2.966 | 0.00302 |
| scale(DistFence) | -0.437 | 0.080 | -5.479 | 4.27E-08 |
| scale(DistWater) | -0.977 | 0.082 | -11.887 | < 2E-16 |
| scale(rain20k) | -0.534 | 0.040 | -13.297 | < 2E-16 |
| scale(Human20) | 0.098 | 0.017 | 5.811 | 6.23E-09 |
| scale(Carbon20_1) | 0.208 | 0.040 | 5.19 | 2.10E-07 |
| scale(evi20k) | 0.113 | 0.028 | 3.973 | 7.10E-05 |
| scale(FP_20k) | -0.322 | 0.052 | -6.169 | 6.86E-10 |
| scale(OWL_20) | 0.227 | 0.033 | 6.948 | 3.69E-12 |
| scale(Bush_20k) | 0.272 | 0.028 | 9.891 | < 2E-16 |
| scale(RoadDens20k) | -0.279 | 0.030 | -9.342 | < 2E-16 |
| scale(TC__20k_) | 0.080 | 0.032 | 2.492 | 0.0127 |

Table S8. Habitat suitability model results showing variable coefficients for wild dog (Lycaon pictus) presence data derived from camera trap (CT) and spoor datasets from across the Kavango Zambezi Transfrontier Conservation Area (KAZA TFCA). Details on model fits and combining datasets for ensemble habitat suitability maps are provided in the main text. Table S1 outlines the variables used for modelling. Variables followed by a number indicate the scale at which that particular variable was most influential ranging from 250m, 500m, 1k, 2.5k, 5k, 7.5k, 10k, or 20k.

| CT | | | | |
| --- | --- | --- | --- | --- |
| Coefficients: | |  |  |  |
|  | Estimate | Std. | Error | z |
| (Intercept) | -3.125 | 0.239 | -13.083 | < 2E-16 |
| scale(x) | -0.526 | 0.098 | -5.347 | 8.96E-08 |
| scale(y) | 0.190 | 0.100 | 1.901 | 0.05729 |
| scale(Human7_5k) | -1.555 | 0.361 | -4.296 | 1.74E-05 |
| scale(Carbon20k) | 0.210 | 0.096 | 2.179 | 0.02934 |
| scale(Water_20) | -0.508 | 0.117 | -4.333 | 1.47E-05 |
| scale(WoodlDen_4) | 0.123 | 0.066 | 1.872 | 0.06117 |
| scale(DistWater) | -0.137 | 0.080 | -1.702 | 0.08883 |
| scale(DistVil) | 0.225 | 0.074 | 3.039 | 0.00237 |
| Effort | 0.028 | 0.004 | 7.218 | 5.28E-13 |
|  |  |  |  |  |
| SPOOR | | | | |
| Coefficients: | |  |  |  |
|  | Estimate | Std. | Error | z |
| (Intercept) | -6.067 | 0.215 | -28.168 | < 2E-16 |
| scale(x) | 0.124 | 0.227 | 0.547 | 0.58406 |
| scale(y) | 0.417 | 0.169 | 2.465 | 0.0137 |
| scale(DistWater) | 1.117 | 0.256 | 4.423 | 9.76E-06 |
| scale(rain20k) | -0.550 | 0.201 | -2.735 | 0.00624 |
| scale(DistFence) | -1.117 | 0.208 | -5.377 | 7.56E-08 |
| scale(Human20) | -1.327 | 0.414 | -3.204 | 0.00136 |
| scale(Carbon2__1) | 0.063 | 0.086 | 0.725 | 0.46819 |
| scale(evi7_5k) | -0.179 | 0.084 | -2.12 | 0.03398 |
| Effort | 0.237 | 0.090 | 2.645 | 0.00817 |
| scale(FP_1k) | -0.322 | 0.178 | -1.813 | 0.06982 |
| scale(Bush_500) | 0.223 | 0.057 | 3.894 | 9.87E-05 |
| scale(WoodlDe_13) | -0.518 | 0.496 | -1.045 | 0.29623 |

Table S9. Habitat suitability model results showing variable coefficients for leopard (Panthera pardus) presence data derived from camera trap (CT) and spoor datasets from across the Kavango Zambezi Transfrontier Conservation Area (KAZA TFCA). Details on model fits and combining datasets for ensemble habitat suitability maps are provided in the main text. Table S1 outlines the variables used for modelling. Variables followed by a number indicate the scale at which that particular variable was most influential ranging from 250m, 500m, 1k, 2.5k, 5k, 7.5k, 10k, or 20k.

| CT | | | | |
| --- | --- | --- | --- | --- |
| Coefficients: | |  |  |  |
| # | Estimate | Std. | Error | z |
| (Intercept) | -1.225 | 0.178 | -6.878 | 6.06E-12 |
| scale(x) | -0.202 | 0.076 | -2.674 | 0.00749 |
| scale(y) | 0.304 | 0.070 | 4.36 | 1.30E-05 |
| scale(Human2_5) | -0.230 | 0.077 | -2.967 | 0.003 |
| scale(evi_2020) | -0.200 | 0.068 | -2.922 | 0.00348 |
| scale(FP_7_5) | 0.149 | 0.067 | 2.224 | 0.02616 |
| scale(Water_1) | -0.425 | 0.073 | -5.809 | 6.27E-09 |
| scale(Crop_7_5) | -0.156 | 0.087 | -1.783 | 0.07459 |
| scale(OpenSh_4) | -0.384 | 0.081 | -4.72 | 2.36E-06 |
| Effort | 0.026 | 0.003 | 7.974 | 1.54E-15 |
|  |  |  |  |  |
| SPOOR | | | | |
| Coefficients: | |  |  |  |
|  | Estimate | Std. | Error | z |
| (Intercept) | -3.824 | 0.083 | -45.873 | < 2E-16 |
| scale(x) | 0.305 | 0.092 | 3.285 | 0.00102 |
| scale(y) | 0.236 | 0.058 | 4.098 | 4.17E-05 |
| scale(DistWater) | 0.249 | 0.078 | 3.192 | 0.00141 |
| scale(Carbon50_1) | 0.103 | 0.037 | 2.771 | 0.00558 |
| scale(TC__20k_) | 0.026 | 0.049 | 0.524 | 0.60039 |
| scale(Bush_5k) | -0.154 | 0.038 | -4.062 | 4.86E-05 |
| scale(OWL_20) | -0.168 | 0.052 | -3.217 | 0.00129 |
| Effort | 0.177 | 0.042 | 4.201 | 2.66E-05 |
| scale(Crop_20) | -0.406 | 0.084 | -4.836 | 1.32E-06 |
| scale(Water_20) | 0.006 | 0.029 | 0.199 | 0.84239 |
| scale(FP_7_5) | -0.444 | 0.055 | -8.141 | 3.91E-16 |
| scale(evi5k) | -0.075 | 0.037 | -2.036 | 0.04171 |
| scale(Human) | -0.227 | 0.082 | -2.774 | 0.00554 |

Table S10. Habitat suitability model results showing variable coefficients for lion (Panthera leo) presence data derived from camera trap (CT) and spoor datasets from across the Kavango Zambezi Transfrontier Conservation Area (KAZA TFCA). Details on model fits and combining datasets for ensemble habitat suitability maps are provided in the main text. Table S1 outlines the variables used for modelling. Variables followed by a number indicate the scale at which that particular variable was most influential ranging from 250m, 500m, 1k, 2.5k, 5k, 7.5k, 10k, or 20k.

| CT | | | | |
| --- | --- | --- | --- | --- |
| Coefficients: |  |  |  |  |
|  | Estimate | Std. | Error | z |
| (Intercept) | -1.765 | 0.212 | -8.338 | < 2E-16 |
| scale(x) | 0.250 | 0.078 | 3.211 | 0.00132 |
| scale(y) | 0.208 | 0.108 | 1.924 | 0.05433 |
| scale(TC20k_) | 0.199 | 0.113 | 1.763 | 0.07795 |
| scale(Rain20k) | -0.362 | 0.083 | -4.354 | 1.34E-05 |
| scale(Human2_5) | -0.753 | 0.258 | -2.923 | 0.00346 |
| scale(Carbon2_5k) | -0.431 | 0.074 | -5.799 | 6.66E-09 |
| scale(Bush_500) | -0.147 | 0.063 | -2.349 | 0.01881 |
| scale(DistVil) | 0.144 | 0.060 | 2.401 | 0.01637 |
| scale(DistWater) | -0.746 | 0.102 | -7.329 | 2.32E-13 |
| Effort | 0.015 | 0.004 | 4.151 | 3.30E-05 |
|  |  |  |  |  |
| SPOOR | | | | |
| Coefficients: |  |  |  |  |
|  | Estimate | Std. | Error | z |
| (Intercept) | -3.927 | 0.125 | -31.34 | < 2E-16 |
| scale(x) | 0.399 | 0.093 | 4.284 | 1.84E-05 |
| scale(y) | -0.110 | 0.074 | -1.482 | 0.13836 |
| scale(Carbon1k_1) | -0.172 | 0.055 | -3.151 | 0.00163 |
| scale(Human) | -0.192 | 0.076 | -2.541 | 0.01106 |
| Effort | 0.104 | 0.061 | 1.713 | 0.08677 |
| scale(evi10k) | -0.008 | 0.066 | -0.122 | 0.9027 |
| scale(DistWater) | -0.403 | 0.092 | -4.368 | 1.25E-05 |
| scale(FP_20k) | -0.145 | 0.073 | -1.982 | 0.04746 |
| scale(DistVill) | -0.098 | 0.076 | -1.291 | 0.19674 |
| scale(Crop_7_5) | -0.172 | 0.068 | -2.527 | 0.01152 |

Residual spatial autocorrelation was assessed using Moran’s I for all models (Table S10). Values ranged from low to moderate across species (Moran’s I ≈ 0.05–0.21), indicating that spatial structure was reduced compared to initial models but not eliminated. This suggests that the models accounted for a substantial proportion of spatial dependence, while some residual spatial structure remained.

Variation partitioning revealed consistent but species-specific patterns in the relative contribution of environmental predictors and spatial coordinates. Across all species, environmental predictors explained a larger proportion of deviance than spatial coordinates when considering their independent effects. The independent contribution of spatial coordinates was consistently small (generally <4%), indicating that model performance was not primarily driven by geographic structure.

However, the relative importance of shared variation between environmental predictors and spatial coordinates varied among species. In several cases (all but Zebra camra trap), environmental predictors showed strong independent effects with minimal spatial contribution, indicating clear ecological relationships. In other species, e.g. Elephants, Lion, Spotted Hyaena, Wild Dog and Zebra SPOOR data a larger shared component indicated that environmental predictors were spatially structured, reflecting underlying environmental gradients across the landscape. A few species exhibited moderate independent spatial contributions e.g. Camera trap data for Buffalo, Zebra and Buffalo and Elephant spor model, suggesting the influence of unmeasured environmental variables or fine-scale spatial processes.

Table S11. Moran’s I results indicating spatial autocorrelation before and after accounting for xy coordinates in all species habitat suitability models. Variance partitioning revealed the extent to which patterns observed by environmental variables alone, spatial variables alone and where these two may intersect (shared).

| Species | Moran’s I pre xy | Moran’s I post xy | Environment (%) | Space (%) | Shared (%) |
| --- | --- | --- | --- | --- | --- |
| Camera Trap Data | | | | | |
| Buffalo | 0.32 | 0.21 | 7.2 | 2.2 | 2.7 |
| Elephant | 0.2 | 0.17 | 14.9 | 3.9 | 0.6 |
| Leopard | 0.15 | 0.1 | 5.2 | 0.8 | 1 |
| Lion | 0.12 | 0.09 | 9.9 | 0.8 | 1.2 |
| Spotted Hyaena | 0.13 | 0.12 | 14 | 1.4 | 1.2 |
| Wild dog | 0.14 | 0.09 | 6.1 | 1.5 | 0.8 |
| Zebra | 0.25 | 0.19 | 9.9 | 0.8 | 15 |
| Spoor Data | | | | | |
| Buffalo | 0.23 | 0.2 | 4.1 | 0.3 | 5.8 |
| Elephant | 0.23 | 0.2 | 4.8 | 0.2 | 19.9 |
| Leopard | 0.08 | 0.08 | 2.4 | 0.2 | ~0.0 |
| Lion | 0.06 | 0.05 | 2.4 | 0.2 | 5.8 |
| Spotted Hyaena | 0.09 | 0.08 | 3.9 | 0.3 | 7.1 |
| Wild dog | 0.13 | 0.12 | 4.1 | 0.3 | 5.8 |
| Zebra | 0.19 | 0.17 | 9.2 | 1.1 | 12.7 |

***Species Specific Connectivity***

For connectivity surfaces, the thresholds for dispersal were set according to available literature (see Table S12). To develop source points for the analysis, data was compiled from various sources related to large herbivore and large carnivore censuses in each partner state, as well as for KAZA as a whole ^7–23^. Estimates for predator densities were supplemented with local unpublished results from WildCRU, WildEntrust, Zambia Carnivore Programme and the Hyena Specialist Group.

When we considered individual species level, there were a few areas that stood out as critical for certain species (Figure S1 - S5). For wild dog, the Khaudum-Ngamiland and Kwando WDAs support critical habitat outside protected areas, and the Kwando WDA is vital for connectivity to what is potentially highly suitable habitat in Angola. The Hwange-Makgadikgadi-Nxai Pans WDA maintains critical habitat for elephant, lion and zebra outside protected areas, and the Zambezi and Kwando regions support critical habitat and potential connectivity for buffalo. In terms of movement away from the core towards the more distant parts of KAZA, lion, spotted hyaena and elephant have the strongest potential connectivity towards Sebungwe, Angola, Khaudum and Kafue. Of all the species analysed, zebra are most restricted to the core of KAZA, with the Hwange-Kazuma-Chobe and Hwange-Makgadikgadi-Nxai Pans being the most important connected habitat. While leopards show low connectivity potential to Kafue and Sebungwe, they have additional highly suitable cores in these regions.

Table S12. Dispersal distances for each focal species wild dog (*Lycaon pictus*), leopard (*Panthera pardus*), lion (*Panthera leo*), spotted hyaena (*Crocuta crocuta*), buffalo (*Syncerus caffer*), zebra (*Equus burchelli)* and elephant (*Loxodonta africana*) to set unit costs thresholds in UNICOR. KDE = kernel density analysis and LCP = least cost paths. KDE and LCP values represent the unit costs used in the analysis that are directly comparable to the units cost rationale (km) for each species’ dispersal.

| Species | KDE | LCP | Unit costs rationale | References |
| --- | --- | --- | --- | --- |
| Lion | 2,000,000 | 10,000,000 | 261 km dispersal | ^24,25^ |
| Spotted Hyaena | 1,750,000 | 10,000,000 | 350 km dispersal | Zach Mills and S. Periquet pers. comment |
| Leopard | 750,000 | 4,038,462 | 105 km dispersal | ^26^ |
| Wild dog | 1,250,000 | 13,461,538 | 350 km dispersal | ^27^ |
| Elephants | 1,250,000 | 9,615,385 | 250 km migration | ^28^ |
| Zebra | 2,125,000 | 11,538,462 | 300 km migration | ^29^ |
| Buffalo | 1,250,000 | 9,615,385 | 250 km migration | ^30^ |

Figure S1. Species-specific resistance to movement, in the Kavango Zambezi Transfrontier Conservation Area (KAZA TFCA), converted from respective habitat suitability for a) wild dog (*Lycaon pictus*); b) leopards (*Panthera pardus*); c) lions (*Panthera leo*); d) spotted hyaenas (*Crocuta crocuta*); e) buffalo (*Syncerus caffer*); f) zebras (*Equus quagga*); g) elephants (*Loxodonta africana*). Darker colours identify higher resistance (lower suitability)

Figure S2. Species-specific cumulative kernel density estimates (KDE) in the Kavango Zambezi Transfrontier Conservation Area (KAZA TFCA) highlighting each species’ core areas (yellow) for movement for a) wild dog (*Lycaon pictus*); b) leopards (*Panthera pardus*); c) lions (*Panthera leo*); d) spotted hyaenas (*Crocuta crocuta*); e) buffalo (*Syncerus caffer*); f) zebras (*Equus quagga*); g) elephants (*Loxodonta africana*). Cold colours denote low connectivity, and warmer patches define core areas.

Figure S3. Species-specific least-cost path (LCP) in the Kavango Zambezi Transfrontier Conservation Area (KAZA TFCA) highlighting each species’ dispersal corridors (yellow) for a) wild dog (*Lycaon pictus*); b) leopards (*Panthera pardus*); c) lions (*Panthera leo*); d) spotted hyaenas (*Crocuta crocuta*); e) buffalo (*Syncerus caffer*); f) zebras (*Equus quagga*); g) elephants (*Loxodonta africana*). Blue indicates low probability of use and yellow high probability of use.

Figure S4. Species-specific core areas (80th percentile of cumulative resistant kernel density estimate (KDE), in red) overlaid on respective least cost-paths (LCP) with blue indicating corridors with low probability of use and yellow corridors with high probability of use in the Kavango Zambezi Transfrontier Conservation Area (KAZA TFCA). Species include a) wild dog (*Lycaon pictus*); b) leopards (*Panthera pardus*); c) lions (*Panthera leo*); d) spotted hyaenas (*Crocuta crocuta*); e) buffalo (*Syncerus caffer*); f) zebras (*Equus quagga*); g) elephants (*Loxodonta africana*).

Figure S5. Species-specific predicted corridors in the Kavango Zambezi Transfrontier Conservation Area (KAZA TFCA) with above average likely usage (50th percentile least cost path (LCP)) for a) wild dog (*Lycaon pictus*); b) leopards (*Panthera pardus*); c) lions (*Panthera leo*); d) spotted hyaenas (*Crocuta crocuta*); e) buffalo (*Syncerus caffer*); f) zebras (*Equus quagga*); g) elephants (*Loxodonta africana*).

***Surrogacy***

Table S12. Correlation coefficients for cumulative resistant kernel (KDE) predicted results, where we examined the overlap in predicted cores for all species including wild dog (*Lycaon pictus*), leopards (*Panthera pardus*), lions (*Panthera leo*), spotted hyaenas (*Crocuta crocuta*), buffalo (*Syncerus caffer*), zebras (*Equus quagga*) and elephants (*Loxodonta africana*) outside the protected area network in the Kavango-Zambezi Transfrontier Conservation Area (KAZA TFCA). In bold we highlight coefficients above 0.65, indicating higher surrogacy potential.

|  | Multispecies | Zebra | Wild dog | Spotted hyaena | Lion | Leopard | Buffalo | Elephant |
| --- | --- | --- | --- | --- | --- | --- | --- | --- |
| Multispecies | 1.000 |  |  |  |  |  |  |  |
| Zebra | **0.809** | 1.000 |  |  |  |  |  |  |
| Wild dog | 0.036 | -0.183 | 1.000 |  |  |  |  |  |
| Spotted Hyaena | **0.803** | 0.592 | -0.277 | 1.000 |  |  |  |  |
| Lion | **0.853** | **0.752** | -0.210 | 0.665 | 1.000 |  |  |  |
| Leopard | 0.363 | 0.193 | 0.025 | 0.152 | 0.504 | 1.000 |  |  |
| Buffalo | **0.746** | 0.598 | -0.047 | 0.500 | 0.549 | 0.172 | 1.000 |  |
| Elephant | **0.790** | 0.646 | -0.232 | **0.735** | **0.679** | 0.123 | 0.470 | 1.000 |

Table S13. Correlation coefficients for where we examined the overlap in least cost paths (LCPs) for all species including wild dog (*Lycaon pictus*), leopards (*Panthera pardus*), lions (*Panthera leo*), spotted hyaenas (*Crocuta crocuta*), buffalo (*Syncerus caffer*), zebras (*Equus quagga*) and elephants (*Loxodonta africana*) outside the protected area network in the Kavango-Zambezi Transfrontier Conservation Area (KAZA TFCA). In bold we highlight coefficients above 0.65, indicating higher surrogacy potential.

|  | Multispecies | Elephant | Leopard | Spotted hyaena | Buffalo | Lion | Wild dog | Zebra |
| --- | --- | --- | --- | --- | --- | --- | --- | --- |
| Multispecies | 1.00 |  |  |  |  |  |  |  |
| Elephant | 0.36 | 1.00 |  |  |  |  |  |  |
| Leopard | 0.41 | 0.31 | 1.00 |  |  |  |  |  |
| Spotted hyaena | 0.31 | 0.30 | 0.26 | 1.00 |  |  |  |  |
| Buffalo | 0.17 | 0.08 | 0.09 | 0.13 | 1.00 |  |  |  |
| Lion | 0.21 | 0.27 | 0.17 | 0.21 | 0.08 | 1.00 |  |  |
| Wild dog | 0.45 | 0.24 | 0.26 | 0.18 | 0.09 | 0.13 | 1.00 |  |
| Zebra | 0.14 | 0.30 | 0.16 | 0.21 | 0.07 | 0.25 | 0.14 | 1.00 |

***Conservation value outside protected areas***

Figure S6. Gaps in protections of highly suitable habitat (hHS, represented by the 80th percentile of habitat suitability results) in the Kavango Zambezi Transfrontier Conservation Area (KAZA TFCA) KAZA. These results indicate the percentage of hHS that falls within each designed wildlife dispersal area in KAZA, per focal species wild dog (*Lycaon pictus*), leopards (*Panthera pardus*), lions (*Panthera leo*), spotted hyaenas (*Crocuta crocuta*), buffalo (*Syncerus caffer*), zebras (*Equus quagga*) and elephants (*Loxodonta africana*).

Figure S7. Average intensity of least cost paths (LCPs), representing gaps in protections of species’ dispersal movement corridors the Kavango Zambezi Transfrontier Conservation Area (KAZA TFCA) wildlife dispersal areas, which are outside the protected area network. The species of focus included wild dog (*Lycaon pictus*), leopards (*Panthera pardus*), lions (*Panthera leo*), spotted hyaenas (*Crocuta crocuta*), buffalo (*Syncerus caffer*), zebras (*Equus quagga*) and elephants (*Loxodonta africana*).

**Discussion**

***Model Spatial Autocorrelation***

Our results demonstrate that, although spatial autocorrelation is present in species occurrence data, environmental predictors remain the primary drivers of model outcomes across species. The inclusion of spatial coordinates reduced residual spatial autocorrelation, supporting their role in accounting for broad-scale spatial structure. However, residual Moran’s I values indicate that some fine-scale spatial dependence remains, which is common in ecological datasets and may reflect unmeasured environmental factors or spatially structured ecological processes.

Importantly, variation partitioning showed that the independent contribution of spatial coordinates was consistently small relative to that of environmental predictors. This indicates that the inclusion of coordinates does not mask ecological signal but instead accounts for residual spatial structure while preserving meaningful habitat relationships.

The magnitude of shared variation between environmental predictors and spatial coordinates varied among species, highlighting differences in how species respond to spatially structured environmental gradients. For some species, the shared component dominated, indicating that environmental effects are strongly spatially organized. For example, with buffalo, zebra and elephant, shared environmental-spatial variation may be reflected by the spatial structuring of rivers and water sources on which they are dependent ^31^. This is particularly pronounced in the dry season, and as rivers or water sources are themselves spatially autocorrelated, this may explain higher overlap between environmental and spatial components.

For others, environmental predictors showed stronger independent effects, suggesting more direct relationships with habitat variables. These differences likely reflect species-specific ecological traits, such as habitat specialization, mobility, and sensitivity to landscape heterogeneity. Overall, these findings indicate that species distributions are shaped by a combination of environmental and spatial processes, with environmental predictors playing a primary role and spatial structure contributing secondarily in a species-dependent manner. While residual spatial autocorrelation remains, it does not invalidate model inference but suggests that results should be interpreted with appropriate caution.

***Species specific results***

Interestingly, while protected areas like national parks and game reserves are often prioritised when it comes to conservation resources, our analyses show that for some species, investment in non-protected areas may be pivotal to species conservation plans and with a view to maintaining connectivity ^32^. Although we were focused on hHS regions, looking solely at areas with HS above the 80th percentile, these results are still worrying concerning potential gaps in the protection of hotspots of suitability for charismatic species in KAZA. For all focal species, less than 20% of hHs (72,666 km2) lies within formally recognised parks and reserves. This is particularly pertinent for wild dogs, for which only 1% of what is currently hHs is under formal protection in KAZA. As an example, the Kwando and Khaudum-Ngamiland WDAs stood out as the area most important for wild dog core habitat, and the Kwando WDA as the most critical from a habitat suitability and connectivity point of view. This is corroborated by other connectivity models based on empirical wild dog movement ^33^, and recent studies that show wild dog presence in the Khaudum-Ngamiland region ^34^. These WDAs should be a key focal area for wild dog protection and population restoration within KAZA, especially in Angola which is currently developing its conservation and tourism plans for Luengwe-Luiana.

Conversely, lions have the highest overlap between hHs and protected areas, with just over 30%. This may be driven by habitat types – a large portion of parks are composed of wetland or savanna systems, which are more appealing habitats for photographic tourism, and in which lions thrive ^35^. More plausibly, this could be explained by the fact that, lions are not tolerated outside protected areas; lions are highly sensitive to settlement and agriculture and because of persecution are less likely to survive outside protected areas ^25,36,37^. For this reason, it is not surprising that the Hwange-Kazuma-Chobe and the Hwange-Makgadikgadi-Nxai Pans WDAs, show the highest habitat suitability for lions in KAZA, as these areas also have the lowest density of people, highest biomass of large prey and are composed largely of wildlife-centric land use types including wildlife management areas and controlled hunting areas ^15,38^. Conservation potential for lions is thus highest in this region. However, lions can move long distances, and as most of the peripheral populations in KAZA outside this core are too small to be self-sustaining 7, connectivity is also critical. Currently, it seems plausible that connectivity from the core to the Kafue in Zambia, Sebungwe region in Zimbabwe and Khaudum NP in Namibia can be maintained. However, there is currently no clear connectivity to Angola. This likely stems from the high density of people in the Zambezi region of Namibia. Despite the designation of corridors in this region, continued utilization threatens to cut off connectivity. Conservation efforts to maintain pathways for animals across this region is critical not only for lions but all other species.

The WDAs with the highest hHS for spotted hyaenas are those that make up the spotted hyena’s continuous core areas including the Sebungwe region in Zimbabwe, Hwange-Kazuma-Chobe from northwestern Zimbabwe to Botswana, and the Zambezi-Chobe-Floodplain and Mosi-oa-Tunya WDAs in southern Zambia. Like lions, these areas coincide with higher numbers of large prey and lower densities of people, both of which are favourable for supporting spotted hyaena (Searle et al. 2023). While spotted hyaenas can be cryptic and exhibit behavioural plasticity that allows them to adapt to some degree of human activity (Green & Holekamp, 2019)), they are heavily persecuted in some areas for livestock depredation ^41^ and may be more sensitive to disturbance than previously thought ^42^. As habitat generalists ^43^, it appears that prey availability and land use type are the most critical factors in spotted hyaena density and persistence ^42,44^. Despite being modelled with one of the lower dispersal thresholds (but see Beytell, 2024 for an exceptional dispersal event), potential for connectivity to the distal PAs in KAZA is high. This is likely facilitated by the persistence of spotted hyaenas at low densities throughout the KAZA landscape. As a persecuted carnivore with low potential recovery post-perturbation ^46,47^, conservation activities for spotted hyaenas should focus on human-predator conflict mitigation that allows resident clans to persist outside protected areas across the KAZA region.

Similarly, leopards are habitat generalists ^48^, and probably the most adaptable large carnivore ^49^. Across KAZA, leopards were detected more than any other large carnivore. In addition to lack of clear habitat selection patterns, leopards are also generalist feeders, allowing them to adapt to a range of prey species ^50^ and persist in areas where smaller prey species are available, as is the case in many of the WDAs outside of protected areas ^38,51^. The Sebungwe WDA in Zimbabwe was identified as an area of hHS for leopards, similarly to all other species. This is likely due to the highest densities of leopards in Matusadona National Park ^52^. However, the neighbouring areas like Chete, Chirisa and Chizarira were found to support lower densities of leopards, probably due to high levels of snaring (for both leopards and prey), hunting and human appropriation of habitat ^52^. To add to this pressure, some studies have also shown that in areas where adult males are removed due to high persecution or due to trophy hunting, young males may become adaptively philopatric, increasing inbreeding risk considerably ^53^. This highlights once more the need to ensure sufficient prey availability and to minimize the effects of human-induced pressures ^51,52^. From a connectivity perspective, there is limited potential for dispersal northwards from the Botswana core. While there is evidence of genetic similarities between Kafue leopards and those in the Hwange-Chobe-Okavango core ^54^, it is likely that these two populations will continue to diverge; the Zambezi River clearly acts as a dispersal barrier and increasing human populations in the Zambezi region poses an additional challenge. In fact, this may already have caused genetic divergence between Botswana, Namibia and Angola leopards, which are geographically closer in proximity ^54^. As leopards can persist in areas with small prey and higher human influence, conservation efforts in the Zambezi Chobe Floodplain and areas surrounding Kafue should focus on human leopard coexistence to ensure long-term population viability of the Kafue leopard stronghold and foster links to Sioma Ngwezi and Angola to the south-west.

For the large herbivores: buffalo, zebra and elephant, which are all water-dependent ^55–57^, our results show highly suitable habitat spread along the Zambezi, Kwando and Kavango River channels, with the most connected areas again in the Botswana core along the Hwange-Kazuma-Chobe WDA. These HS results are corroborated by the findings of the KAZA Elephant Aerial Survey ^15^ for all three species. The limitation of habitat suitability to waterways is particularly evident for buffalo. For buffalo, the Zambezi-Mosi-oa-Tunya and Zambezi-Chobe Floodplain WDAs rank highly for proportion of hHS. While buffalo are known to use the former, the latter is more likely to be functional as a seasonal corridor rather due to the high density of people in this WDA ^58^. For the zebra, the Hwange-Kazuma-Chobe and Hwange-Makgadikgadi-Nxai Pans are critical hHS areas and support two important migrations from Chobe to Nxai Pan and the Okavango to Makgadikgadi ^29,55^. These routes are also highlighted in the 80th percentile connectivity paths for zebra, supporting the accuracy of our predictive models in this area. Zebra connectivity seems limited across the Zambezi River, and so connectivity along the panhandle towards Angola through the Kwando WDA could be critical for restoring zebra migration routes into Angola and Sioma Ngwezi. However, the veterinary fences once again limit this potential. Lastly, for elephants, much like lions, the Hwange-Kazuma-Chobe and Hwange-Makgadikgadi-Nxai Pans are critical habitats, where there are also lower numbers of people ^15^. For all three species, connectivity appears to be limited by a) the Zambezi River and b) the buffalo fences along the Namibia/Botswana boundary leading towards Zambia and Angola, in agreement with other studies in KAZA ^57,59,60^. The removal of the veterinary fences and the intensive protection of corridors in the Zambezi region could go a long way in re-establishing corridors for large herbivores in a critical transboundary of the Kwando WDA.

References

1.	Karger, D. N., Wilson, A. M., Mahony, C., Zimmermann, N. E. & Jetz, W. Global daily 1 km land surface precipitation based on cloud cover-informed downscaling. *Sci. Data* **8**, 307 (2021).

2.	Spawn, S., Sullivan, C., Lark, T. & Gibbs, H. Harmonized global maps of above and belowground biomass carbon density in the year 2010. *Sci. Data* **7**, 112 (2020).

3.	Hansen, M. C. *et al.* High-Resolution Global Maps of 21st-Century Forest Cover Change. *Science (1979).* **342**, (2013).

4.	Inman, V. & Lyons, M. Automated Inundation History Mapping over Large Areas using Landsat and Google Earth Engine. https://www.preprints.org/manuscript/202003.0038 (2020).

5.	Wolski, P., Murray-Hudson, M., Thito, K. & Cassidy, L. Keeping it simple: Monitoring flood extent in large data-poor wetlands using MODIS SWIR data. *International Journal of Applied Earth Observation and Geoinformation* **57**, 224–234 (2017).

6.	Gebhardt, S. *Land Cover Classification Applying Sentinel Imagery for Kavango Zambezi Transfrontier Conservation Area (KAZA TFCA).* (2021).

7.	Chase, M. J. *et al.* Continent-wide survey reveals massive decline in African savannah elephants. *PeerJ* **2016**, e2354 (2016).

8.	Loveridge, A., Seymour-Smith, J. L., Sibanda, A. & Sousa, L. L. *Interim Report on Large Predator Camera Trap Surveys in Western and Northern Zimbabwe*. (2019).

9.	Dunham, K. M., Mackie, C. S., Nyaguse, G. & Zhuwau, C. *Aerial Survey of Elephants and Other Large Herbivores in North-West Matabeleland (Zimbabwe)*. (2015).

10.	Dunham, K. M., Mackie, C. S., Nyaguse, G. & Zhuwau, C. *Aerial Survey of Elephants and Other Large Herbivores in the Sebungwe (Zimbabwe) 2014*. (2015).

11.	Funston, P., Beytell, P., Hanssen, L., Singwangwa, M. & Tchadau, B. A. *Status Report Lions of the Kavango and Zambezi Regions*. (2017).

12.	Hanssen, L., Singwnagwa, M. & Kukuwa, V. *Camera Trap Survey to Determine the Status of Leopards in the Mudumu-North Complex, Zambezi Region, Namibia*. (2015).

13.	Funston, P. *et al.* *The Distribution and Sttus of Lions and Other Large Carnviores in Luenge-Luiana and Mavinga National Parks, Angola.* . (2017).

14.	Potgieter, G., Thomson, R., Doden, E. & Winterbach, C. W. W. *Large Carnivores and Their Prey in Western Ngamiland, Botswana*. (2017).

15.	Bussière, E. & Potgieter, D. *KAZA Elephant Survey 2022*. (2023).

16.	MEFT, LCMAN & NCE. *Conservation Status and Red List of the Terrestrial Carnivores of Namibia*. (Windhoek, Namibia, 2022).

17.	MEFT. *Wildlife Census of Namibia’s North-East Rivers*. (2022).

18.	Chase, M., Schlossberg, S., Sutcliffe, R. & Seonyatseng, E. *Dry Season Aerial Survey of Elephants and Wildlife in Northern Botswana, July-October 2018*. (2019).

19.	Young, K. *et al.* *Large Carnivore Survey Sioma Ngwezi National Park*. (2015).

20.	DNPW. *Report on Aerial Survey of Elephant and Large Terrestrial Herbivores in the Kafue and Sioma-Ngwezi Ecosystems 2019*. (2019).

21.	Panthera & Department of National Parks and Wildlife. *Lion Monitoring Report Kafue National Park 2018-2022*. (2023).

22.	Panthera & Department of National Parks and Wildlife. *Leopard Monitoring Report Greater Kafue Ecosystem 2018-2022*. (2023).

23.	DNPW. *Report of Aerial Survey of Elephants and Other Large Herbivores in the Kafue National Park and Its Surrounding Management Areas 2021*. (2021).

24.	Loveridge, A. J., Sousa, L. L., Cushman, S., Kaszta, Ż. & Macdonald, D. W. Where have all the lions gone? Establishing realistic baselines to assess decline and recovery of African lions. *Divers. Distrib.* **28**, (2022).

25.	Elliot, N. B., Cushman, S. A., Macdonald, D. W. & Loveridge, A. J. The devil is in the dispersers: Predictions of landscape connectivity change with demography. *Journal of Applied Ecology* **51**, 1169–1178 (2014).

26.	Wilkinson, A. *et al.* Leopard dispersal across a fragmented landscape in the Western Cape, South Africa. *Afr. J. Ecol.* **62**, e13284 (2024).

27.	Cozzi, G. *et al.* African Wild Dog Dispersal and Implications for Management. *Journal of Wildlife Management* **84**, 614–621 (2020).

28.	Purdon, A., Mole, M., Chase, M. & van Aarde, R. J. Partial migration in savanna elephant populations distributed across southern Africa. *Sci. Rep.* 11331 (2018) doi:10.1038/s41598-018-29724-9.

29.	Naidoo, R., Chase, M., Beytell, P., Du Preez, P. & Landen, K. A newly discovered wildlife migration in Namibia and Botswana is the longest in Africa. *Oryx* https://doi.org/10.1017/S0030605314000222 (2016) doi:10.1017/S0030605314000222.

30.	Naidoo, R., Du Preez, P., Stuart-Hill, G., Jago, M. & Wegmann, M. Home on the range: Factors explaining partial migration of African buffalo in a tropical environment. *PLoS One* **7**, (2012).

31.	Naidoo, R. *et al.* Mapping and assessing the impact of small-scale ephemeral water sources on wildlife in an African seasonal savannah. *Ecological Applications* **30**, (2020).

32.	Khosravi, R. , Hemami, M. R. & Cushman, S. A. Multispecies assessment of core areas and connectivity of desert carnivores in central Iran. *Divers. Distrib.* **24**, 193–207 (2018).

33.	Hofmann, D. D. *et al.* A three-step approach for assessing landscape connectivity via simulated dispersal: African wild dog case study. *Landsc. Ecol.* **38**, 981–998 (2023).

34.	Kotze, R. *et al.* *Western Ngamiland Camera Trap Survey Report* . (2025).

35.	Nicholson, S. *et al.* *Panthera Leo. The IUCN Red List of Threatened 2023: E.T15951A231696234.* https://dx.doi.org/10.2305/IUCN.UK.2023-1.RLTS.T15951A231696234.en (2023).

36.	Loveridge, A. J., Valeix, M., Elliot, N. B. & Macdonald, D. W. The landscape of anthropogenic mortality: how African lions respond to spatial variation in risk. *Journal of Applied Ecology* **54**, 815–825 (2017).

37.	Van der Weyde, L. K., Mbisana, C. & Klein, R. Multi-species occupancy modelling of a carnivore guild in wildlife management areas in the Kalahari. *Biol. Conserv.* **220**, (2018).

38.	Winterbach, H. E. K., Winterbach, C. W. & Somers, M. J. Landscape suitability in Botswana for the conservation of its six large African carnivores. *PLoS One* **9**, 1–12 (2014).

39.	Green, D. S., Johnson-Ulrich, L., Couraud, H. E. & Holekamp, K. E. Anthropogenic disturbance induces opposing population trends in spotted hyenas and African lions. *Biodivers. Conserv.* **27**, 871–889 (2018).

40.	Green, D. & Holekamp, K. Pastoralist activities affect the movement patterns of a large African carnivore, the spotted hyena (Crocuta crocuta). *J. Mammal.* **100**, 1941–1953 (2019).

41.	Bohm, T. & Höner, O. R. *Crocuta Crocuta. The IUCN Red List of Threatened Species 2015: E.T5674A45194782.* https://dx.doi.org/10.2305/IUCN.UK.2015-2.RLTS.T5674A45194782.en. (2015).

42.	Searle, C. E. *et al.* Spotted hyaena population density across habitat and land use types in southern Tanzania. *J. Zool.* **322**, 89–100 (2024).

43.	Holekamp, K. E. & Dloniak, S. M. Intraspecific variation in the behavioral ecology of a tropical carnivore, the spotted hyena. in *Advances in the Study of Behaviour* vol. 42 189–229 (Academic Press, 2010).

44.	Briers-Louw, W. D. *et al.* Estimated baseline density of a spotted hyaena population in a post-war landscape. *Oryx* 1–11 (2025).

45.	Beytell, P., Hanssen, L., Aschenborn, O. & Naidoo, R. Long‐Distance, Transfrontier Carnivore Dispersals in Southern Africa. *Ecol. Evol.* **14**, e70754 (2024).

46.	Benhaiem, S. *et al.* Slow recovery from a disease epidemic in the spotted hyaena, a keystone social carnivore. *Communications Biology* **1**, 1–12 (2018).

47.	Becker, M. S. *et al.* Wire-snare bushmeat poaching and the large African carnivore guild: Impacts, knowledge gaps, and field-based mitigation. *Biol. Conserv.* **289**, 110376 (2024).

48.	Leedham, S., Paijmans, J. L., Manica, A. & Leonardi, M. Niche conservatism in a generalist felid: low differentiation of the climatic niche among subspecies of the leopard (Panthera pardus). *bioRxiv* **2023**, (2023).

49.	Stein, A. B. *et al.* *Panthera Pardus (Amended Version of 2019 Assessment). The IUCN Red List of Threatened Species 2020: E.T15954A163991139*. https://dx.doi.org/10.2305/IUCN.UK.2020-1.RLTS.T15954A163991139.en (2020).

50.	Hayward, M. W. *et al.* Prey preferences of the leopard (Panthera pardus). *J. Zool.* **270**, 298–313 (2006).

51.	Searle, C. E. *et al.* Drivers of leopard (Panthera pardus) habitat use and relative abundance in Africa’s largest transfrontier conservation area. *Biol. Conserv.* **248**, (2020).

52.	Loveridge, A. J., Sousa, L. L., Seymour-Smith, J. L., Mandisodza-Chikerema, R. & Macdonald, D. W. Environmental and anthropogenic drivers of African leopard Panthera pardus population density. *Biol. Conserv.* **272**, (2022).

53.	Fattebert, J., Balme, G., Dickerson, T., Slotow, R. & Hunter, L. Density-dependent natal dispersal patterns in a leopard population recovering from over-harvest. *PLoS One* **10**, 1–15 (2015).

54.	Thacker, T. A genetic perspective on leopard (Panthera pardus) conservation units across southern Africa. (University of Cape Town, Cape Town, 2021).

55.	Bartlam-Brooks, H. L. A., Beck, P. S. A., Bohrer, G. & Harris, S. In search of greener pastures: Using satellite images to predict the effects of environmental change on zebra migration. *J. Geophys. Res. Biogeosci.* **118**, 1427–1437 (2013).

56.	Bennitt, E., Bonyongo, M. C. & Harris, S. Habitat selection by African buffalo (Syncerus caffer) in response to landscape-level fluctuations in water availability on two temporal scales. *PLoS One* **9**, 1–14 (2014).

57.	Naidoo, R. *et al.* Landscape connectivity for African elephants in the world’s largest transfrontier conservation area: A collaborative, multi-scalar assessment. *Journal of Applied Ecology* **61**, 2483–2496 (2024).

58.	Loveridge, A. *et al.* *Chobe Zambezi Floodplain Wildlife Dispersal Area Integrate Land Use and Management Project*. (2023).

59.	Naidoo, R. *et al.* Challenges to Elephant Connectivity From Border Fences in the World’s Largest Transfrontier Conservation Area. *Frontiers in Conservation Science* **3**, (2022).

60.	Brennan, A. *et al.* Characterizing multispecies connectivity across a transfrontier conservation landscape. *Journal of Applied Ecology* **57**, 1700–1710 (2020).
